# Interferon-λ signaling to maternal dendritic cells protects against congenital Zika virus infection

**DOI:** 10.1126/sciadv.aeh1073

**Published:** 2026-10-23

**Authors:** Margaret R. Dedloff, Thomas R. Reinhardt, Tessa-Jonne F. Ropp, Joel Rivera-Cardona, Celeste R. Robles, Rebecca L. Casazza, J. Ashley Ezzell, Michelle S. Itano, Helen M. Lazear

**Affiliations:** ^1^ Department of Microbiology & Immunology, University of North Carolina at Chapel Hill, Chapel Hill, NC, USA.; ^2^ Neuroscience Microscopy Core Facility, Carolina Institute for Developmental Disabilities & Neuroscience Center, University of North Carolina at Chapel Hill, Chapel Hill, NC, USA.; ^3^ Histology Research Core Facility, Department of Cell Biology & Physiology, University of North Carolina at Chapel Hill, Chapel Hill, NC, USA.; ^4^ Department of Cell Biology & Physiology, University of North Carolina at Chapel Hill, Chapel Hill, NC, USA.

## Abstract

Interferon-λ (IFN-λ, type III IFN) mediates antiviral immunity at anatomic barriers, including the maternal-fetal interface. To investigate the effects of IFN-λ during congenital Zika virus (ZIKV) infection, we infected mice lacking the IFN-αβ receptor (*Ifnar1*^−/−^) or both the IFN-αβ and IFN-λ receptors (*Ifnar1*^−/−^*Ifnlr1*^−/−^) at embryonic day 9 and found that loss of maternal IFN-λ signaling resulted in greater transplacental transmission, highlighting a protective effect of IFN-λ at the maternal-fetal interface. To investigate the spatial relationship between ZIKV infection and antiviral responses, we used HiPlex RNAscope on the entire gravid uterus and found that IFN-λ was expressed more proximal to the site of ZIKV infection in *Ifnar1*^−/−^ dams compared to *Ifnar1*^−/−^*Ifnlr1*^−/−^ dams. We performed immunophenotyping of the placenta and uterus by flow cytometry and found a decrease in dendritic cells and natural killer (NK) cells in the uterus of *Ifnar1*^−/−^*Ifnlr1*^−/−^ dams compared to *Ifnar1*^−/−^ dams, but NK cell depletion did not affect fetal infection. Using conditional knockout mice, we identified maternal dendritic cells as the key IFN-λ–responsive cell type mediating protection against ZIKV congenital infection. Together, our data support a model in which IFN-λ signaling to maternal dendritic cells increases dendritic cell accumulation in the uterus, which facilitates a protective antiviral response that restricts ZIKV congenital infection.

## INTRODUCTION

Only a subset of pathogens, including Zika virus (ZIKV), rubella virus, and human cytomegalovirus, can cross the placental barrier during pregnancy and cause congenital infections (*1*, *2*). Congenital infections can result in a range of birth defects and adverse pregnancy outcomes, such as fetal loss and intrauterine growth restriction (IUGR). Furthermore, the adverse effects of congenital infections can manifest as cognitive and functional disabilities later in childhood, even in infants seemingly unaffected at birth (*3*–*5*). ZIKV is a mosquito-borne orthoflavivirus that usually presents with fever and rash, but the 2015 to 2017 ZIKV outbreak in the Americas revealed the ability of ZIKV to cause congenital Zika syndrome when infection occurs during pregnancy (*6*–*10*). Congenital Zika syndrome is observed in ∼6% of pregnancies with ZIKV infection and encompasses a spectrum of fetal abnormalities including IUGR, microcephaly, and stillbirth (*6*–*9*).

Immunity at the maternal-fetal interface is a complex balance between protecting the fetus from maternal pathogens and safeguarding against maternal immune-mediated rejection of the semiallogeneic fetus and placenta (*11*–*13*). The placenta is a fetal-derived tissue that invades the maternal endometrium (the decidua), forming the interface between maternal and fetal tissues during pregnancy. Nutrients are passed from the mother to the fetus through the placental villi, the surface of which is formed by syncytiotrophoblasts, which are specialized cells fused to form a single-cell barrier between maternal and fetal blood. Syncytiotrophoblasts are highly resistant to infection by diverse pathogens, making them an important barrier against fetal infection (*14*–*16*). Conversely, tolerogenic immune mechanisms at the maternal-fetal interface prevent maternal immune rejection of the fetus (*17*). This delicate balance between protective and tolerogenic immunity changes over the course of gestation (*18*).

Interferon-λ (IFN-λ, type III IFN) is a cytokine that induces a similar antiviral transcriptional program as type I IFNs (IFN-αβ) but is especially important at anatomic barriers, including the maternal-fetal interface (*19*, *20*). In contrast to the ubiquitous expression of the IFN-αβ receptor (heterodimer of IFNAR1 and IFNAR2), the IFN-λ receptor (heterodimer of IFNLR1 and IL10Rb) is found predominantly on epithelial cells and a subset of immune cells, including myeloid cells (*21*–*30*). Both IFN-αβ and IFN-λ have immunomodulatory properties, but IFN-λ is thought to be less potent and inflammatory compared to IFN-αβ (*31*–*39*). During pregnancy, IFN-λ is constitutively secreted by syncytiotrophoblasts (*23*, *40*) and decidual cells (*41*). IFN-λ is produced in response to ZIKV infection in trophoblasts in cell culture and primary decidual tissues (*40*, *42*), and treatment of mice with IFN-λ during infection decreases ZIKV viral loads (*42*, *43*). Previously, we demonstrated that IFN-λ acts specifically through signaling in maternal tissues (rather than fetal/placental tissues) following ZIKV infection during pregnancy, but the effects of IFN-λ can be either pathogenic or protective depending on when infection occurs during gestation (*44*). IFN-λ reduced ZIKV transplacental transmission when infection occurred later in pregnancy [embryonic day 9 (E9)]; in contrast, IFN-λ signaling induced fetal pathology when infection occurred earlier in pregnancy (E7). Using poly(I:C) treatment to mimic the IFN-inducing effects of viral infection, we found that the pathogenic effects of IFN-λ during early gestation occurred through IFN-λ signaling in maternal leukocytes (*44*), but the maternal cell types responsible for IFN-λ–mediated protection against transplacental transmission remain undefined.

Here, we further define the protective role for IFN-λ during congenital ZIKV infection. Using a mouse model of congenital ZIKV infection, we demonstrate that maternal IFN-λ signaling is protective against fetal infection and transplacental transmission. We found that ZIKV infection occurs in fetuses, placentas, and the uterus regardless of maternal IFN-λ signaling, but there is a more robust antiviral response near the site of infection in dams with intact IFN-λ signaling. We found that dams with intact IFN-λ signaling have an increased proportion and number of natural killer (NK) cells at the maternal-fetal interface during congenital ZIKV infection, but these cells were not responsible for IFN-λ–mediated protection against fetal infection and transplacental transmission. We show that dendritic cells accumulate at the maternal-fetal interface during congenital ZIKV infection in an IFN-λ–dependent manner, and we demonstrate that IFN-λ signaling to dendritic cells is required for protection against fetal infection and transplacental transmission. Together, our results suggest that IFN-λ plays both immunomodulatory and antiviral roles at the maternal-fetal interface during congenital ZIKV infection and that maternal dendritic cells are key IFN-λ–responsive cells at this site.

## RESULTS

### Maternal IFN-λ signaling protects against fetal ZIKV infection in *Ifnar1*^−/−^ mice

Our group and others previously showed that maternal IFN-λ signaling protects against fetal infection when ZIKV infection occurs later in gestation (E9 and E12) (*42*, *44*). However, these experiments used wild-type and *Ifnlr1*^−/−^ mice treated with an IFNAR1-blocking monoclonal antibody (mAb), which is sufficient to promote ZIKV infection, but not virus-induced pathology. To assess the protective effects of IFN-λ in the context of a more robust ZIKV infection, we used pregnancies in which dams and fetuses both lack IFN-αβ signaling (*Ifnar1*^−/−^). We set up crosses such that fetuses (and their associated placentas) all retained IFN-λ signaling either in the context of dams that lacked IFN-λ signaling (*Ifnar1*^−/−^ *Ifnlr1*^−/−^ double-knockout) or dams that retained IFN-λ signaling (*Ifnar1*^−/−^) so as to evaluate the effects of IFN-λ signaling specifically in maternal tissues. We evaluated fetal pathology, fetal viral loads, and fetal infection rates in *Ifnar1^−/−^* dams mated with *Ifnar1^−/−^ Ifnlr1^−/−^* sires or *Ifnar1^−/−^ Ifnlr1^−/−^* dams mated with *Ifnar1^−/−^* sires. At 9 days postmating (E9), pregnant dams were inoculated with 1000 focus-forming units (FFU) of ZIKV strain FSS13025 via a subcutaneous injection in the footpad, and maternal and fetal tissues were harvested 6 days postinfection (dpi) (E15) to measure viral loads and assess fetal pathology (Fig. 1A). We found no difference in maternal spleen viral loads between *Ifnar1^−/−^* dams and *Ifnar1^−/−^ Ifnlr1^−/−^* dams (Fig. 1B), but fetuses from dams lacking IFN-λ signaling exhibited significantly higher viral loads compared to dams with intact IFN-λ signaling (median fetal viral loads 3.41 versus 5.06 log_10_ copies/ml, *P* < 0.0001; Fig. 1C). In addition, we found that dams lacking IFN-λ signaling had increased rates of fetal infection compared to dams with intact IFN-λ signaling (97% versus 87% of fetuses positive for ZIKV RNA, *P* < 0.05; Fig. 1D and table S1). We also measured infectious viral titers in the fetuses by plaque assay and found that dams lacking IFN-λ signaling had significantly higher fetal viral loads (median 3.32 versus 3.41 log_10_ plaque-forming units/g, *P* < 0.05; fig. S1A) and fetal infection rates (36% versus 57% of fetuses positive for virus, *P* < 0.05; fig. S1B) compared to dams with intact IFN-λ signaling, consistent with reverse transcription quantitative polymerase chain reaction (RT-qPCR) results. All fetuses were weighed and photographed to evaluate two measurements of fetal pathology: IUGR, defined as fetal weights below 1 SD of the mean of fetuses from mock-infected mice, and resorptions, defined as implantation sites with no discernable fetal or placental structures (Fig. 1, E to G). We found that there was a significant decrease in fetal weights from dams with intact IFN-λ signaling but no differences in fetal pathology between dams with intact IFN-λ signaling and dams that lack IFN-λ signaling, suggesting that increased viral loads in fetuses from dams that lack IFN-λ signaling do not correspond with changes in fetal weights. Overall resorption and IUGR rates were low, likely due to the lack of fetal IFN-αβ signaling, which is required for high rates of fetal pathology, in this experimental design (*44*, *45*).

**Fig. 1.**
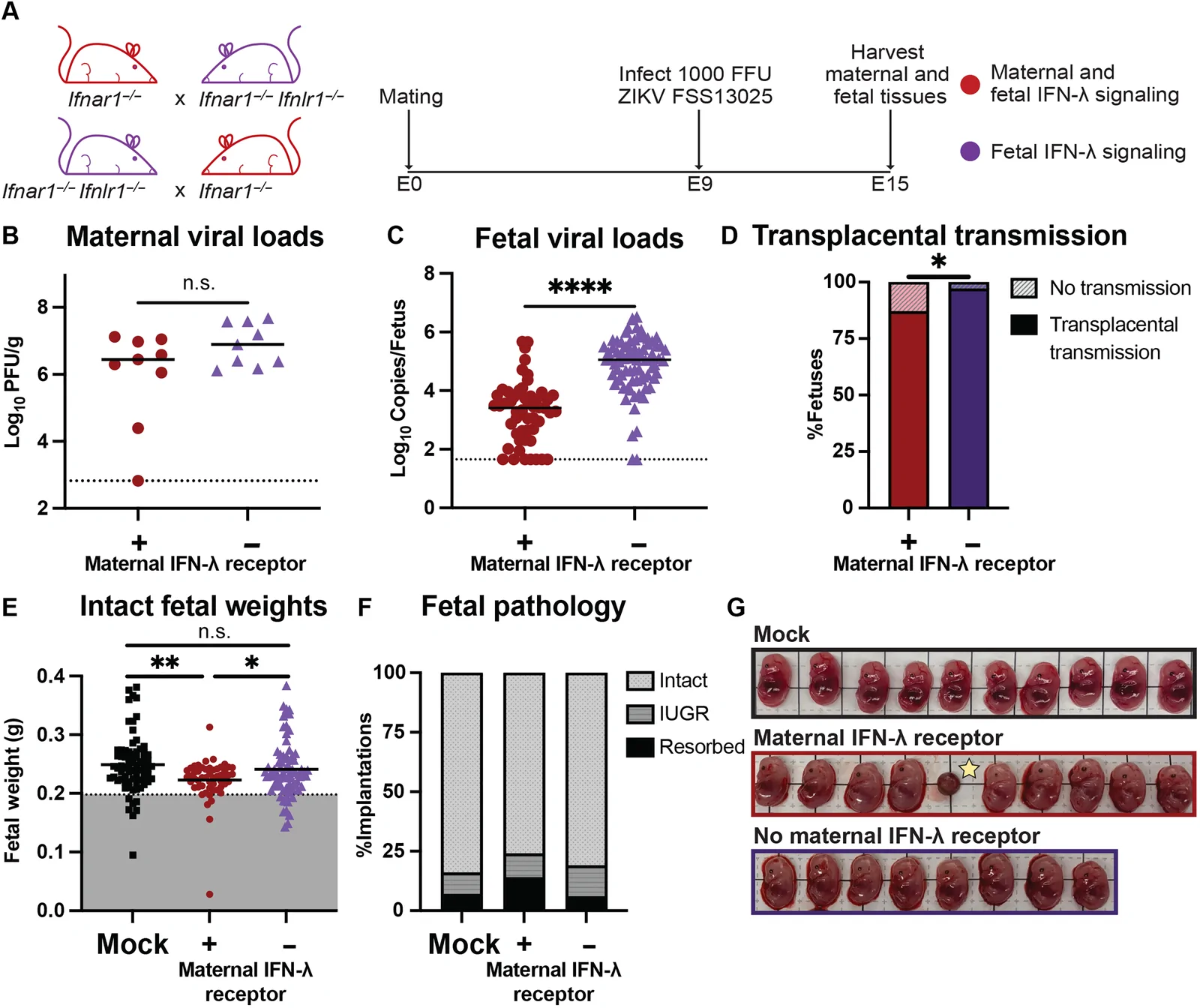
Maternal IFN-λ signaling protects against fetal ZIKV infection in *Ifnar1*^−/−^ mice. (**A**) Mating and infection timeline. Dams from *Ifnar1^−/−^* x *Ifnar1^−/−^ Ifnlr1^−/−^* or *Ifnar1^−/−^ Ifnlr1^−/−^* x *Ifnar1^−/−^* crosses were inoculated at day 9 postmating (E9) with ZIKV FSS13025 by subcutaneous injection in the footpad. Maternal and fetal tissues were harvested at E15. (**B**) Viral loads in the maternal spleen were measured by plaque assay. Each data point represents one dam. PFU, plaque-forming units. (**C**) Fetal viral loads were measured by RT-qPCR. Each data point represents one fetus. (**D**) Transplacental transmission was calculated as the percent of fetuses with viral loads above the limit of detection. (**E**) Intact fetuses were weighed. Dotted line indicates 1 SD below the mean of fetuses from mock-infected dams; fetal weights below this threshold were classified as having IUGR. (**F**) Proportions of implantation sites exhibiting IUGR or resorption. (**G**) Representative images of one pregnancy from each cross. The star indicates resorption. Each square in the grid is 1 cm. Results are combined from 62 fetuses from 9 *Ifnar1*^−/−^ dams, 82 fetuses from 10 *Ifnar1^−/−^ Ifnlr1^−/−^* dams, and 78 fetuses from 9 uninfected dams (5 *Ifnar1*^−/−^ dams and 4 *Ifnar1^−/−^ Ifnlr1^−/−^* dams) from 11 independent experiments. Viral loads were compared by the Mann-Whitney test, proportions by the Cochran-Armitage test, and fetal weights by the *t* test (*P < 0.05; **P < 0.01; ****P < 0.0001; n.s., not significant).

### IFN-λ protects against fetal infection regardless of infection duration

Previously, we found that maternal IFN-λ signaling can have either protective or pathogenic effects depending on when infection occurred during gestation, likely due to differences in placental development at time of infection (*44*). However, these results were based on fetuses harvested at E15, meaning that, when infections occurred at E7, fetuses were harvested 8 dpi, compared to 6 dpi for infections at E9 (fig. S2A), leaving the possibility that the pathogenic effects of IFN-λ with E7 infection only become evident as the infection progressed. To address this question, we evaluated viral loads, infection rates, and pathology of fetuses harvested from dams at E15 (6 dpi) or E17 (8 dpi). We mated dams with intact IFN-λ signaling (*Ifnar1*^−/−^) with sires lacking IFN-λ signaling (*Ifnar1^−/−^ Ifnlr1^−/−^*) or the reverse mating scheme. Dams were inoculated at E9 with 1000 FFU of ZIKV. We found that dams lacking IFN-λ signaling had significantly higher fetal viral loads compared to dams with intact IFN-λ signaling at both 6 dpi (median 3.47 versus 6.80 log_10_ copies/ml, *P* < 0.0001) and 8 dpi (median 4.14 versus 5.49 log_10_ copies/ml, *P* < 0.0001; fig. S2B). Furthermore, at both time points, dams lacking IFN-λ signaling had significantly higher rates of transplacental transmission compared to dams with intact IFN-λ signaling (6 dpi 84% versus 100%, *P* < 0.01; 8 dpi 84% versus 98%, *P* < 0.05; fig. S2C and table S1). Maternal IFN-λ signaling did not affect resorption rate (fig. S2D). Together, these results show that maternal IFN-λ signaling protects against transplacental transmission after E9 ZIKV infection, regardless of infection duration.

### ZIKV infection occurs in fetuses, placentas, and the uterus

Because the architecture of the maternal-fetal interface is complex and unique, determining where viral infection occurs is critical to understanding the pathogenic mechanisms of congenital infections (*1*, *46*, *47*). To determine where ZIKV infection occurs at the maternal-fetal interface, we mated *Ifnar1^−/−^* dams with *Ifnar1^−/−^ Ifnlr1^−/−^* sires and *Ifnar1^−/−^ Ifnlr1^−/−^* dams with *Ifnar1^−/−^* sires, inoculated dams with ZIKV at E9, and harvested tissues at E13 to evaluate the spatial localization of ZIKV RNA in the gravid uterus using HiPlex RNAscope (Fig. 2A). We harvested gravid uteruses at E13 for microscopy analysis to facilitate tissue processing, as E15 uteruses are too large for standard embedding cassettes and require decalcification for high-quality sectioning. We detected ZIKV in fetuses, placentas, and uteruses from *Ifnar1^−/−^* and *Ifnar1^−/−^ Ifnlr1^−/−^* dams (Fig. 2B and fig. S3A). In separate pregnancies, we measured viral loads by RT-qPCR. Dams lacking IFN-λ signaling exhibited significantly higher viral loads in fetuses (median 7.11 versus 6.18 log_10_ copies/g, *P* < 0.0001) and placentas (median 8.20 versus 7.87 log_10_ copies/g, *P* < 0.001) compared to dams with intact IFN-λ signaling (Fig. 2, C and D, and table S1), but there was no significant difference in viral loads in uteruses (Fig. 2E). These findings suggest that maternal IFN-λ signaling does not affect infection in maternal tissues but reduces ZIKV transmission to the placenta and fetus.

**Fig. 2.**
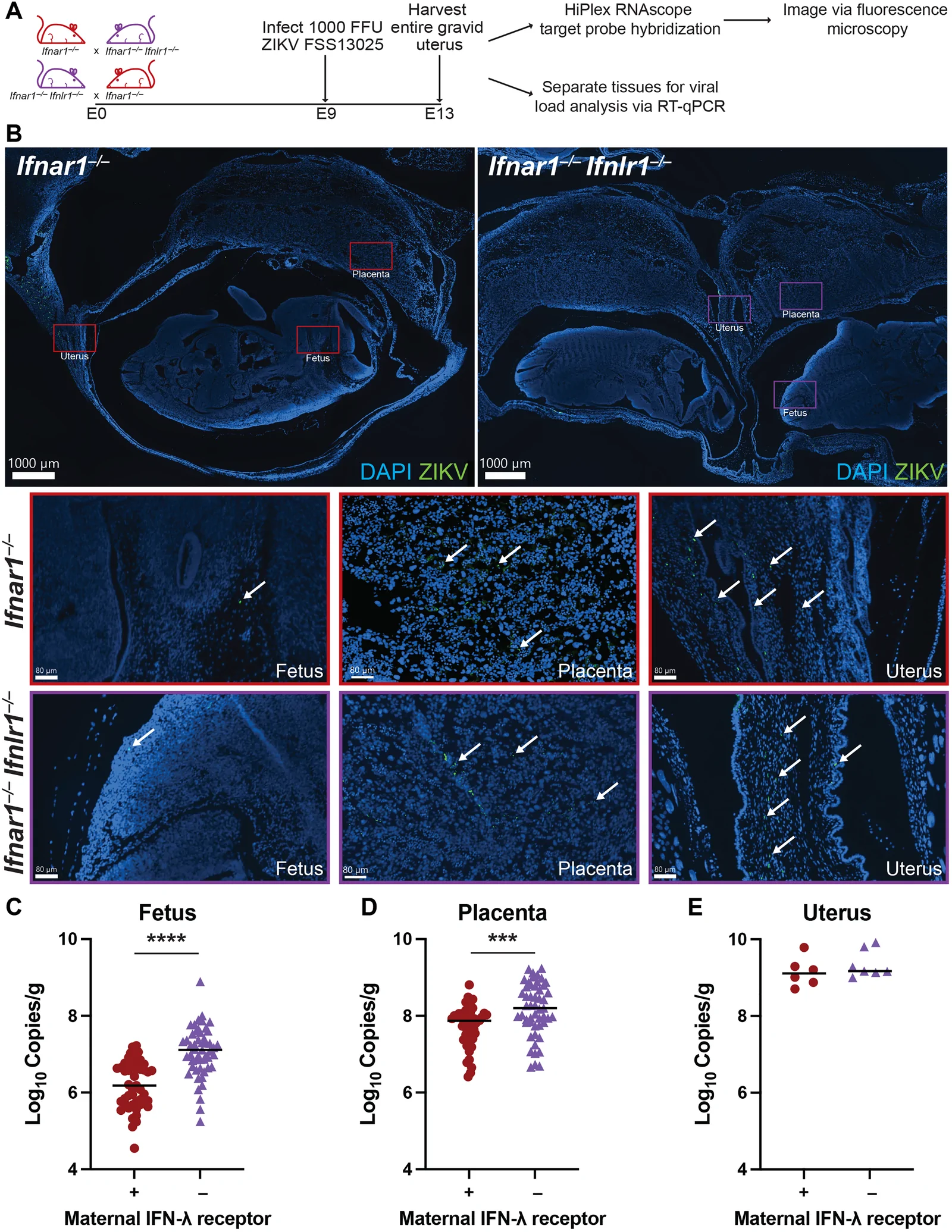
ZIKV infection occurs in fetuses, placentas, and the uterus. (**A**) Mating and infection timeline. Dams from *Ifnar1^−/−^* x *Ifnar1^−/−^ Ifnlr1^−/−^* or *Ifnar1^−/−^ Ifnlr1^−/−^* x *Ifnar1^−/−^* crosses were inoculated at E9 with ZIKV FSS13025 by subcutaneous injection in the footpad. Maternal and fetal tissues were harvested at E13. (**B**) Representative images of portions of implantation sites. Sections of the entire gravid uterus (two *Ifnar1*^−/−^ dams and two *Ifnar1^−/−^ Ifnlr1^−/−^* dams per group, collected in independent experiments; dams from experiment 2 shown) were probed for ZIKV RNA (green) and stained with DAPI (blue). Arrows indicate ZIKV staining. Scale bars in the top row of images are 1000 μm, and those in other images are 80 μm. (**C** to **E**) Viral loads in fetuses, placentas, and uterus were measured by RT-qPCR. Each data point represents one fetus (C), one placenta (D), or one dam (E). Results are combined from 52 fetuses from six *Ifnar1*^−/−^ dams and 58 fetuses from seven *Ifnar1^−/−^ Ifnlr1^−/−^* dams from seven independent experiments. Viral loads were compared by the Mann-Whitney test (***P < 0.001; ****P < 0.0001).

### Maternal IFN-λ signaling increases IFN-λ expression at the site of infection

To investigate the spatial relationship between ZIKV infection and the IFN-λ response, we mated *Ifnar1^−/−^* dams with *Ifnar1^−/−^ Ifnlr1^−/−^* sires and *Ifnar1^−/−^ Ifnlr1^−/−^* dams with *Ifnar1^−/−^* sires, inoculated dams with ZIKV at E9, harvested the entire gravid uterus at E13, and probed for ZIKV and *Ifnl2/3* RNA by HiPlex RNAscope. Using the Imaris microscopy image analysis software, we assigned spots for ZIKV and *Ifnl2/3* and color coded *Ifnl2/3* spots based on the distance to the nearest ZIKV spot (Fig. 3A). We found that *Ifnl2/3* spots were closer to ZIKV spots in dams with intact IFN-λ signaling compared to dams lacking IFN-λ signaling (Fig. 3, B and C; fig. S3B; and table S2). To determine whether the proximity of *Ifnl2/3* expression to ZIKV infection reflected changes in antiviral gene expression across the entire placenta, we mated *Ifnar1^−/−^* dams with *Ifnar1^−/−^ Ifnlr1^−/−^* sires and *Ifnar1^−/−^ Ifnlr1^−/−^* dams with *Ifnar1^−/−^* sires, inoculated dams with ZIKV at E9, harvested placentas at E15, and detected *Ifnl3*, *Ifnb1*, and *Ifit1* expression by RT-qPCR (Fig. 3, D to F). Gene expression was compared to *Actb* and normalized to the baseline gene expression in placentas from uninfected dams. We found no effect of maternal IFN-λ signaling on induction of *Ifnl3*, *Ifnb1*, or *Ifit1* in placenta homogenates, highlighting the importance of the spatial relationship between the IFN-λ–mediated antiviral response and control of ZIKV infection at the maternal-fetal interface.

**Fig. 3.**
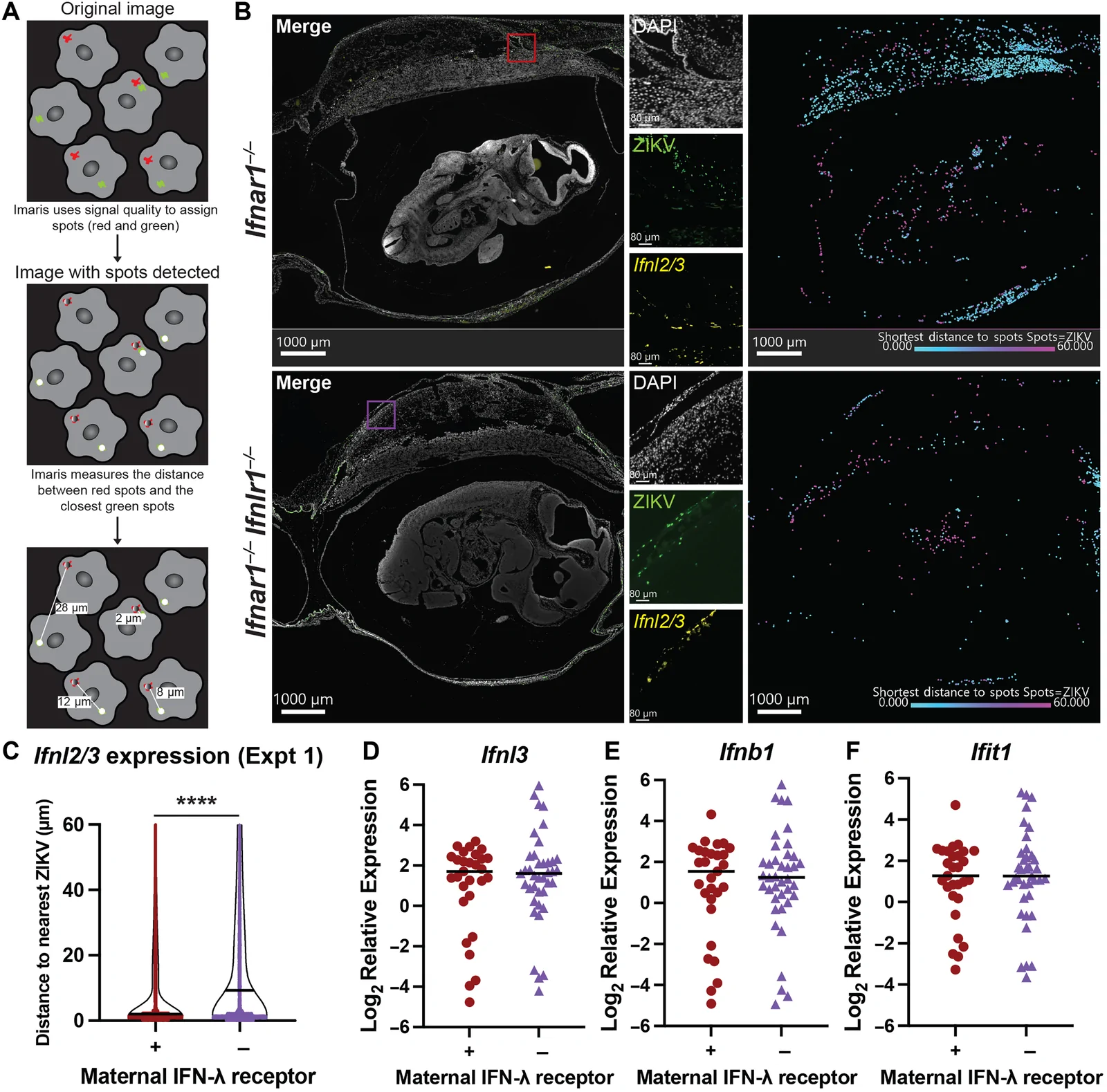
Maternal IFN-λ signaling increases IFN-λ expression at the site of infection. (**A**) Image analysis pipeline. Spots are assigned to signal by the Imaris software, which then measures the distance between spots. (**B**) Representative images of implantation sites. Dams from *Ifnar1^−/−^* x *Ifnar1^−/−^ Ifnlr1^−/−^* or *Ifnar1^−/−^ Ifnlr1^−/−^* x *Ifnar1^−/−^* crosses were inoculated at E9 with ZIKV FSS13025 by subcutaneous injection in the footpad. Gravid uteruses were harvested at E13. Sections of the entire gravid uterus (two *Ifnar1*^−/−^ dams and two *Ifnar1^−/−^ Ifnlr1^−/−^* dams per group, collected in independent experiments, experiment 1 shown) were probed for ZIKV RNA (green) and *Ifnl2/3* RNA (yellow) and stained with DAPI (white). Spots were assigned to ZIKV and *Ifnl2/3* signal. *Ifnl2/3* spots are colored on the basis of their distance to the closest ZIKV spot. Inset image scale bars are 80 μm; other scale bars are 1000 μm. (**C**) Distance of *Ifnl2/3* spots to the nearest ZIKV spot. Each data point represents one *Ifnl2/3* spot. Spots >60 μm from the nearest ZIKV spot are not graphed (*Ifnar1*^−/−^ dam: 3616 spots not graphed of the 23,777 total spots; *Ifnar1^−/−^ Ifnlr1^−/−^* dam: 3544 spots not graphed of the 16,055 total spots). Black horizontal lines indicate the median. Distances were compared by the Mann-Whitney test (****P < 0.0001). (**D** to **F**) Dams were inoculated at E9 with ZIKV FSS13025 by subcutaneous injection in the footpad. Maternal and fetal tissues were harvested at E15. *Ifnl3*, *Ifnb1*, and *Ifit1* expression was measured in placentas by RT-qPCR. Gene expression was compared to *Actb* and normalized to the baseline gene expression in placentas from uninfected dams. Each data point represents one placenta, black lines indicate medians, and data are combined from eight independent experiments. *Ifnar1^−/−^*: 21 placentas from three uninfected dams and 29 placentas from five infected dams; *Ifnar1^−/−^ Ifnlr1^−/−^*: 18 placentas from two uninfected dams and 39 placentas from five infected dams.

### Maternal IFN-λ signaling induces NK cell accumulation in the uterus during congenital ZIKV infection

To determine how maternal IFN-λ signaling regulates immune cell populations at the maternal-fetal interface during congenital ZIKV infection, we used flow cytometry and a broad immunophenotyping panel. We mated *Ifnar1^−/−^* dams with *Ifnar1^−/−^ Ifnlr1^−/−^* sires and *Ifnar1^−/−^ Ifnlr1^−/−^* dams with *Ifnar1^−/−^* sires, inoculated dams with ZIKV at E9, and harvested uteruses and placentas at E15. Comparing *Ifnar1^−/−^* dams to *Ifnar1^−/−^ Ifnlr1^−/−^* dams, we observed some differences in leukocyte populations in placentas, but these differences were either in number of cells or percentage but not both, potentially due to the large variation among placentas in live cell yield and total leukocyte numbers (fig. S4). In the uterus, we found no significant difference in T cells (CD4+ and CD8+), B cells, macrophages, or monocytes between *Ifnar1^−/−^* dams and *Ifnar1^−/−^ Ifnlr1^−/−^* dams; we found a significant decrease in number (but not percentage) of ɣδ T cells (fig. S5). However, we found that the uteruses of dams lacking IFN-λ signaling had a significantly lower percentage (mean 10% versus 15%, *P* < 0.01) and number (mean 876 versus 3039, *P* < 0.001) of NK cells compared to dams with intact IFN-λ signaling (Fig. 4, A to C, and fig. S6). In contrast, dams lacking IFN-λ signaling exhibited a lower percentage but not the number of NK cells in placentas (mean 2.8% versus 1.7%, *P* < 0.0001; mean 94 cells versus 90 cells; Fig. 4, D to F). Together, these results suggest that maternal IFN-λ signaling promotes NK cell accumulation in the uterus during congenital ZIKV infection.

**Fig. 4.**
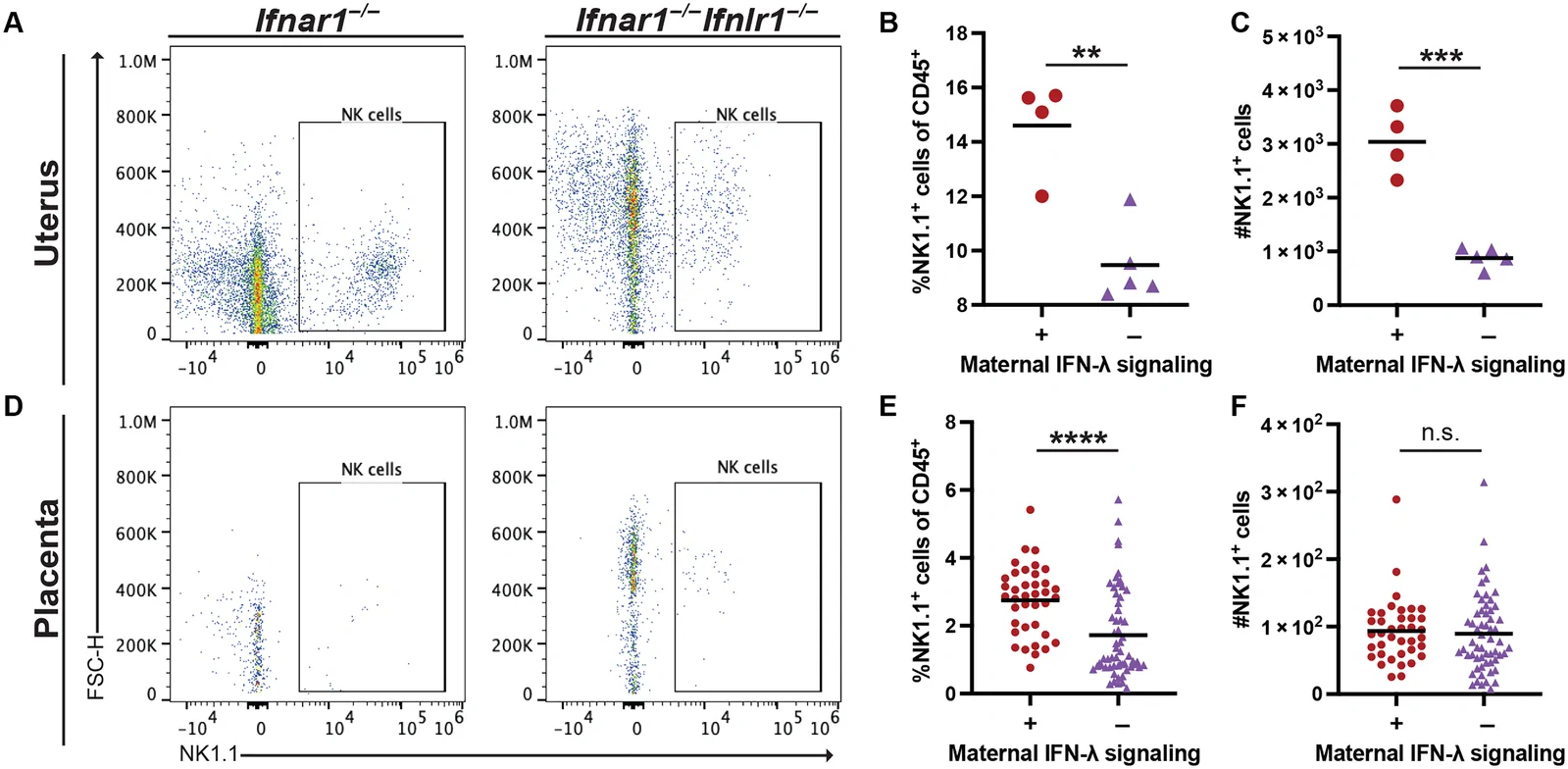
Maternal IFN-λ signaling induces NK cell accumulation in the uterus during congenital ZIKV infection. Dams from *Ifnar1^−/−^* x *Ifnar1^−/−^ Ifnlr1^−/−^* or *Ifnar1^−/−^ Ifnlr1^−/−^* x *Ifnar1^−/−^* crosses were inoculated at E9 with ZIKV FSS13025 by subcutaneous injection in the footpad. (**A**) Gravid uteruses were harvested at E15 and analyzed by flow cytometry (four *Ifnar1*^−/−^ dams and five *Ifnar1^−/−^ Ifnlr1^−/−^* dams collected in four independent experiments). Representative flow cytometry plots of leukocytes stained for NK1.1. (**B** and **C**) Frequency and number of NK cells (NK1.1^+^ out of CD45^+^) in uteruses from *Ifnar1*^−/−^ and *Ifnar1^−/−^ Ifnlr1^−/−^* dams. Groups were compared by the unpaired *t* test (**P < 0.01; ***P < 0.001). (**D**) Placentas were harvested at E15 and analyzed by flow cytometry (38 placentas from five *Ifnar1*^−/−^ dams and 59 placentas from eight *Ifnar1^−/−^ Ifnlr1^−/−^* dams collected in six independent experiments). Representative flow cytometry plots of leukocytes stained for NK1.1. (**E** and **F**) Frequency and number of NK cells (NK1.1^+^ out of CD45^+^) in placentas from *Ifnar1*^−/−^ and *Ifnar1^−/−^ Ifnlr1^−/−^* dams. Groups were compared by the unpaired *t* test (****P < 0.0001; n.s., not significant).

To determine whether IFN-λ–dependent NK cell accumulation at the maternal-fetal interface contributes to protection against fetal infection, we mated *Ifnar1^−/−^* dams with *Ifnar1^−/−^ Ifnlr1^−/−^* sires, inoculated with ZIKV at E9 and depleted NK cells. We treated dams intraperitoneally with 100 μg of αNK1.1 antibody at E9, E11, and E13 and harvested maternal and fetal tissues at E15 to measure fetal viral loads (fig. S7A). We evaluated the efficiency of NK cell depletion systemically and at the maternal-fetal interface using flow cytometry. NK cells were efficiently depleted from both whole blood (mean 3.0% versus 22.6% of leukocytes, *P* < 0.01; fig. S7B) and the spleen (mean 0.1% versus 1.7% of leukocytes, *P* < 0.01; fig. S7C). NK cells were also efficiently depleted in the uterus (mean 3.3% versus 14.6% of leukocytes, *P* < 0.001) and placentas (mean 0.2% versus 2.7% of leukocytes, *P* < 0.0001) in dams that received the αNK1.1 antibody (fig. S7, D and E). Similar effects were observed on NK cell numbers as for percentages (fig. S7F). Despite efficient depletion of NK cells systemically and in tissues at the maternal-fetal interface, we did not observe any difference in fetal viral loads (median 5.40 versus 5.71 log_10_ copies/ml; fig. S7G), suggesting that NK cells are not required for IFN-λ–mediated protection against ZIKV fetal infection.

### IFN-λ signaling promotes dendritic cell infiltration to the uterus during congenital ZIKV infection

When assessing immune cell populations at the maternal-fetal interface, we found that the uteruses of dams lacking IFN-λ signaling had fewer dendritic cells (CD45^+^ and CD11c^hi^) compared to dams with intact IFN-λ signaling as both the percentage of leukocytes (mean 16.1% versus 8.8%, *P* < 0.001) and the number (mean 3292 versus 844 cells, *P* < 0.0001; Fig. 5, A to C). In the placentas, we found an IFN-λ–dependent increase in dendritic cells as percentage of leukocytes (10.2% versus 5.9%, *P* < 0.0001) but no significant difference in dendritic cell number (mean 375 versus 359 cells; Fig. 5, D to F). We used RNAscope to determine the spatial distribution of dendritic cells at the maternal-fetal interface. We harvested tissues at E13, probed for *Itgax* (CD11c), and assigned *Itgax+* spots in Imaris (Fig. 5, G and H; fig. S3C; and table S2). We observed *Itgax* signal largely in the uterus and placentas, with some signal detected in the fetuses. Furthermore, although the spatial distribution of *Itgax* signal did not change between *Ifnar1^−/−^* and *Ifnar1^−/−^ Ifnlr1^−/−^* dams, we found a decrease in *Itgax* signal in *Ifnar1^−/−^ Ifnlr1^−/−^* dams compared to *Ifnar1^−/−^* dams. Together, these results suggest that maternal IFN-λ signaling promotes dendritic cell accumulation at the maternal-fetal interface during congenital ZIKV infection.

**Fig. 5.**
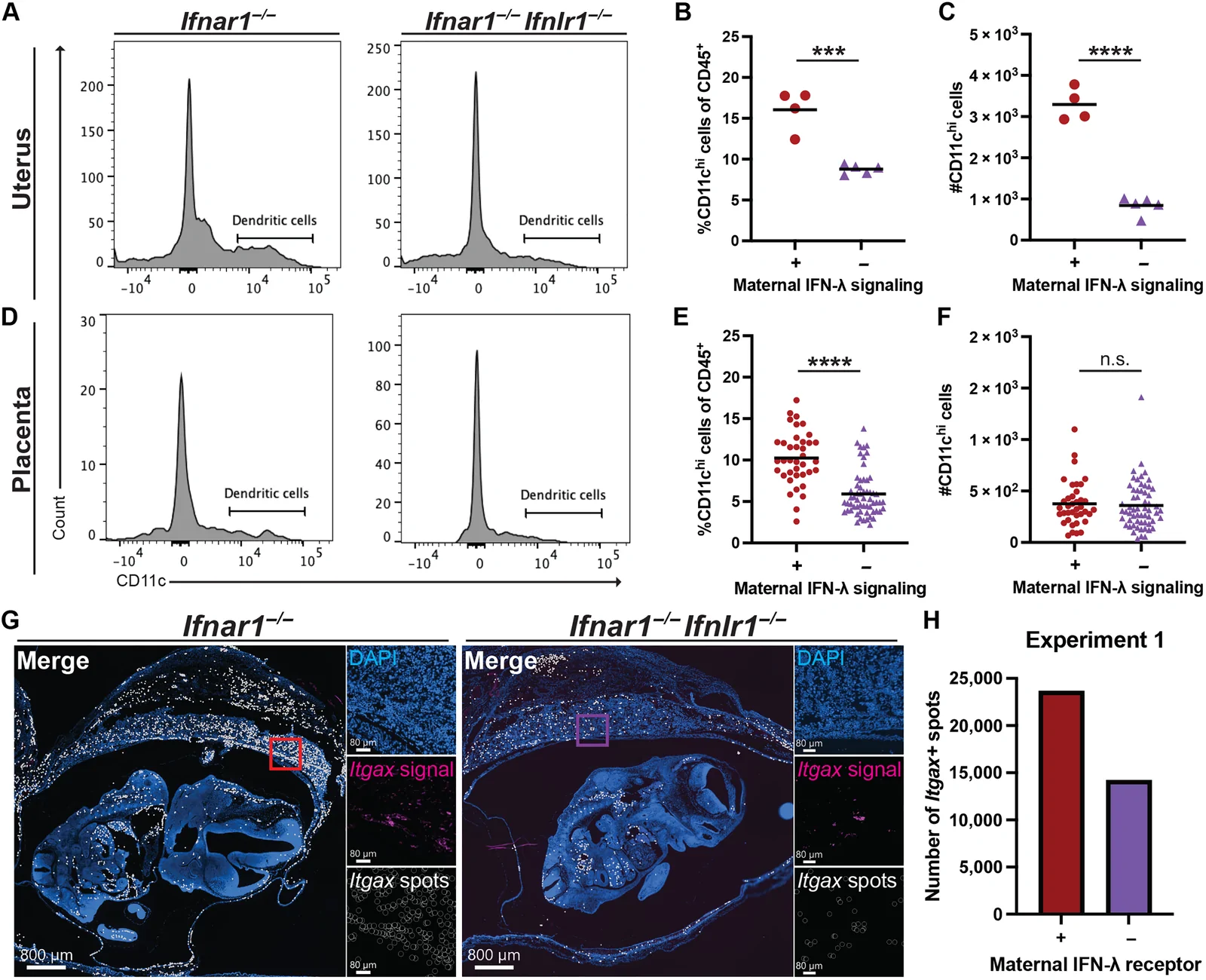
IFN-λ signaling promotes dendritic cell infiltration to the uterus during congenital ZIKV infection. Dams from *Ifnar1^−/−^* x *Ifnar1^−/−^ Ifnlr1^−/−^* or *Ifnar1^−/−^ Ifnlr1^−/−^* x *Ifnar1^−/−^* crosses were inoculated at E9 with ZIKV FSS13025 by subcutaneous injection in the footpad. (**A**) Gravid uteruses were harvested at E15 and analyzed by flow cytometry (four *Ifnar1*^−/−^ dams and five *Ifnar1^−/−^ Ifnlr1^−/−^* dams collected in four independent experiments). Representative flow cytometry plots of leukocytes stained for CD11c in the uterus. (**B** and **C**) Frequency and number of dendritic cells (CD11c^hi^ out of CD45^+^) in uteruses from *Ifnar1*^−/−^ and *Ifnar1^−/−^ Ifnlr1^−/−^* dams. Groups were compared by the unpaired *t* test (***P < 0.001; ****P < 0.0001). (**D**) Placentas were harvested at E15 and analyzed by flow cytometry (38 placentas from five *Ifnar1*^−/−^ dams and 59 placentas from eight *Ifnar1^−/−^ Ifnlr1^−/−^* dams collected in six independent experiments). Representative flow cytometry plots of leukocytes stained for CD11c in placentas. (**E** and **F**) Frequency and number of dendritic cells (CD11c^hi^ out of CD45^+^) in placentas from *Ifnar1*^−/−^ and *Ifnar1^−/−^ Ifnlr1^−/−^* dams. Groups were compared by the unpaired *t* test (****P < 0.0001; n.s., not significant). (**G**) Representative images of implantation sites. Gravid uteruses were harvested at E13. Sections of the entire gravid uterus (two *Ifnar1*^−/−^ dams and two *Ifnar1^−/−^ Ifnlr1^−/−^* dams per group, collected in independent experiments, experiment 1 shown) were probed for *Itgax* RNA (pink), indicating dendritic cells, and stained with DAPI (blue). Spots were assigned on the basis of the quality of the *Itgax* signal (white). Inset image scale bars are 80 μm; other scale bars are 800 μm. (**H**) *Itgax* spots were quantified in each individual microscopy experiment (experiment 1 shown).

### Maternal IFN-λ signaling to dendritic cells protects against transplacental transmission

To determine the IFN-λ–responsive maternal cell type that mediates protection against transplacental transmission, we used conditional knockout dams that lack IFN-λ signaling ubiquitously (*Actin*-Cre-*Ifnlr1^−/−^*), in myeloid cells (*LysM*-Cre-*Ifnlr1^−/−^*), in neutrophils (*Mrp8*-Cre-*Ifnlr1^−/−^*), or dendritic cells (*CD11c*-Cre-*Ifnlr1^−/−^*). These mice were bred as Cre hemizygotes, which results in litters that are 50% Cre+ and 50% Cre−. Experiments were conducted blinded as dams were genotyped following harvesting to allow retrospective comparison of Cre+ and Cre− dams. Conditional knockout dams were mated with wild-type sires and inoculated with ZIKV at E9, 1 day after administration of 2 mg of IFNAR1-blocking mAb MAR1-5A3. Maternal and fetal tissues were harvested at E15 (Fig. 6A). We found no difference in maternal spleen viral loads between Cre− dams and any *Ifnlr1* conditional knockout dams (Fig. 6B), consistent with our findings that IFN-λ signaling does not affect ZIKV viral loads in maternal tissues (Fig. 1B). *Actin*-Cre-*Ifnlr1^−/−^* dams had significantly higher fetal viral loads (median 4.07 versus 1.94 log_10_ copies/ml, *P* < 0.0001; Fig. 6C) and fetal infection rates (86% versus 56%, *P* < 0.0001; Fig. 6D and table S1) compared to Cre− dams, consistent with what we observe in mice on an *Ifnar1^−/−^* background (Fig. 1). Together, these results further support a role for maternal IFN-λ signaling in controlling fetal infection.

**Fig. 6.**
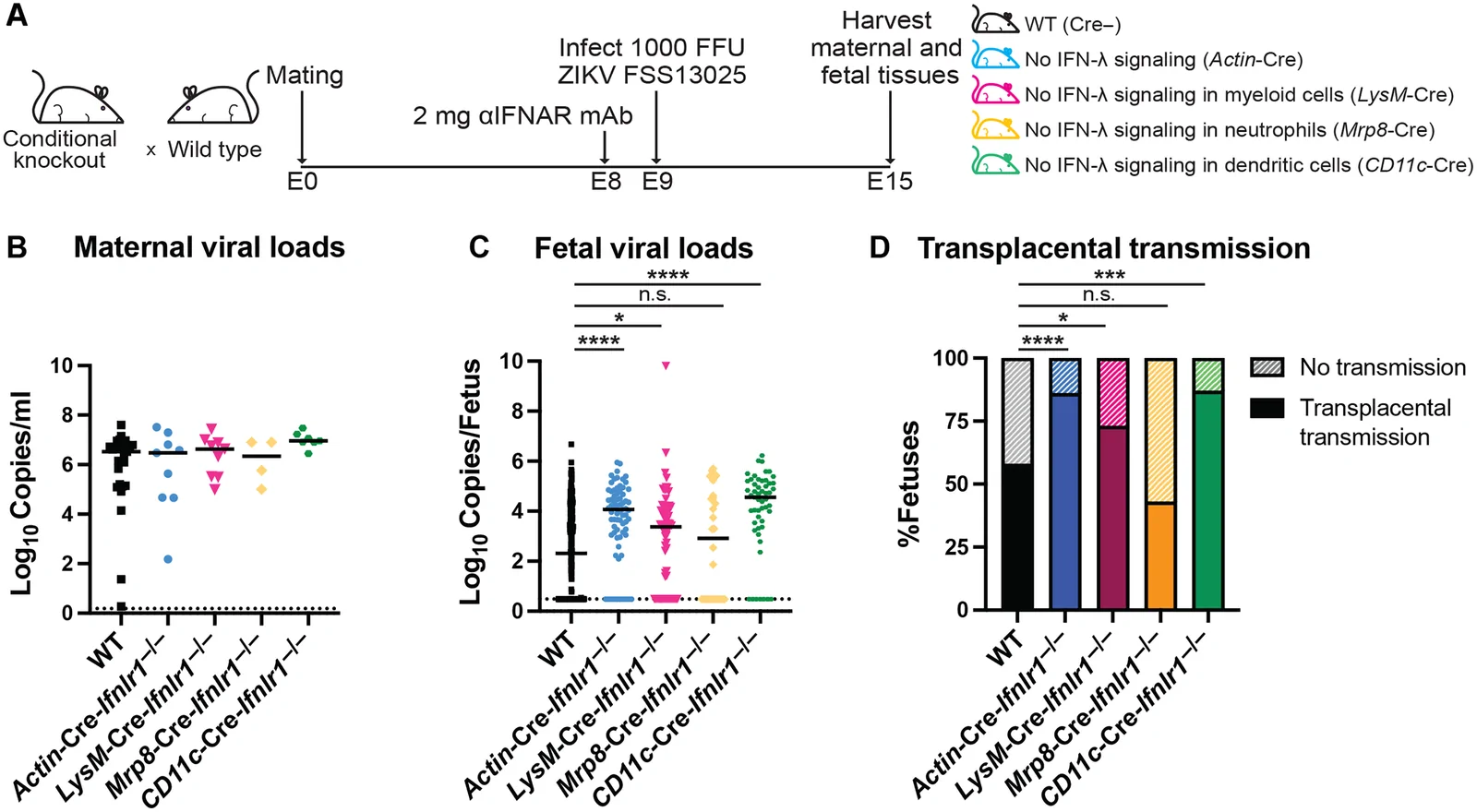
Maternal IFN-λ signaling to dendritic cells protects against transplacental transmission. (**A**) Mating and infection timeline. Dams from conditional knockout x wild-type crosses [227 fetuses from 30 wild-type (WT; Cre−) dams, 82 fetuses from 10 *Actin-*Cre-*Ifnlr1^−/−^* dams, 96 fetuses from 12 *LysM-*Cre-*Ifnlr1^−/−^* dams, 30 fetuses from 4 *Mrp8-*Cre-*Ifnlr1^−/−^* dams, and 53 fetuses from 7 *CD11c-*Cre-*Ifnlr1^−/−^* dams] were treated with 2 mg of anti-IFNAR1 mAb at E8 and inoculated at E9 with ZIKV FSS13025 by subcutaneous injection in the footpad. Maternal and fetal tissues were harvested at E15. (**B** and **C**) Viral loads in maternal spleens and fetuses were measured by RT-qPCR. Each data point represents one dam (B) or one fetus (C). Cre+ groups were compared to Cre− by the Mann-Whitney test (*P < 0.05; ****P < 0.0001; n.s., not significant). (**D**) Transplacental transmission was calculated as the percent of fetuses with viral loads above the limit of detection. Groups were compared by the Cochran-Armitage test (*P < 0.05; ****P < 0.0001; n.s., not significant).

*LysM*-Cre-*Ifnlr1^−/−^* dams exhibited significantly higher fetal viral loads compared to Cre− dams (median 3.37 versus 1.94 log_10_ copies/ml, *P* < 0.05) and similar fetal viral loads compared to *Actin*-Cre-*Ifnlr1^−/−^* dams (median 3.37 versus 4.07 log_10_ copies/ml). Dams lacking IFN-λ signaling in myeloid cells (*LysM*-Cre-*Ifnlr1^−/−^*) exhibited an increased rate of transplacental transmission (73% ZIKV positive) similar to that observed in mice lacking IFN-λ signaling globally, suggesting that IFN-λ signaling to maternal myeloid cells promotes lower fetal viral loads and decreased transplacental transmission. *Mrp8*-Cre-*Ifnlr1^−/−^* dams exhibited no significant difference in fetal viral loads compared to Cre− dams (median 2.91 versus 1.94 log_10_ copies/ml) or fetal infection rates (43% versus 56%), suggesting that neutrophils do not mediate IFN-λ–dependent protection against fetal infection. In contrast, dams lacking IFN-λ signaling in dendritic cells *CD11c*-Cre-*Ifnlr1^−/−^* exhibited increased fetal viral loads (median 4.59 versus 1.94 log_10_ copies/ml, *P* < 0.0001) and fetal infection rates (89% versus 56%, *P* < 0.0001). Fetal viral loads and infection rates in these mice were comparable to those observed in *Actin*-Cre-*Ifnlr1^−/−^* dams, suggesting that maternal dendritic cells are the key IFN-λ–responsive cell type mediating protection against ZIKV congenital infection.

## DISCUSSION

IFN-λ has an important role in combating viral infections at anatomic barriers by inducing an antiviral response, maintaining barrier integrity, and modulating the response of immune cells, together promoting a local response that is less inflammatory and damaging than that of type I IFNs (*33*, *36*, *38*, *39*, *48*–*53*). IFN-λ has a well-documented role at the maternal-fetal interface during congenital infections (*20*, *41*–*44*, *54*, *55*), but the specific cell types that respond to IFN-λ to mediate its protective and pathogenic effects at this site are not defined. To investigate the role of maternal IFN-λ signaling in protection against congenital ZIKV infection, we used a variety of transgenic mouse lines that lack IFN-λ signaling. Because our prior work showed that IFN-λ acted specifically through signaling in maternal tissues (*44*), here we set up pregnancies such that all fetuses and placentas retained IFN-λ signaling, allowing us to focus specifically on the effects of maternal IFN-λ signaling. We found that IFN-λ restricts ZIKV congenital infection as dams that lack IFN-λ signaling (*Ifnar1^−/−^ Ifnlr1^−/−^*) had increased fetal viral loads and transplacental transmission rates, regardless of the duration of the infection. The protective effects of IFN-λ were specific to transplacental transmission because maternal IFN-λ signaling had no impact on infection in maternal tissues, consistent with prior findings demonstrating a lack of effect of IFN-λ on systemic infection (*42*, *44*, *50*, *56*). Although RT-qPCR did not reveal IFN-λ–dependent changes in antiviral gene responses in placenta homogenates, microscopy analysis of whole gravid uteruses probed by HiPlex RNAscope revealed that IFNs were expressed more proximally to sites of ZIKV infection in dams with intact IFN-λ signaling, implying that maternal IFN-λ signaling facilitates a protective antiviral response in the placenta which may restrict viral infection to localized regions of the placenta, preventing transplacental transmission. These findings highlight the importance of spatial relationships between infection and immune responses in the complex architecture of the maternal-fetal interface. Using flow cytometry, we found that a lack of maternal IFN-λ signaling resulted in a decrease in NK cells and dendritic cells in the uterus. Comparison of fetal infection rates in *Ifnlr1^−/−^* conditional knockout mice revealed that maternal myeloid cells, specifically dendritic cells, are key IFN-λ–responsive cell types required for protection against fetal infection. Together, our data support a model in which maternal IFN-λ signaling to dendritic cells increases their accumulation in the uterus and facilitates a protective antiviral response that restricts ZIKV fetal infection (Fig. 7).

**Fig. 7.**
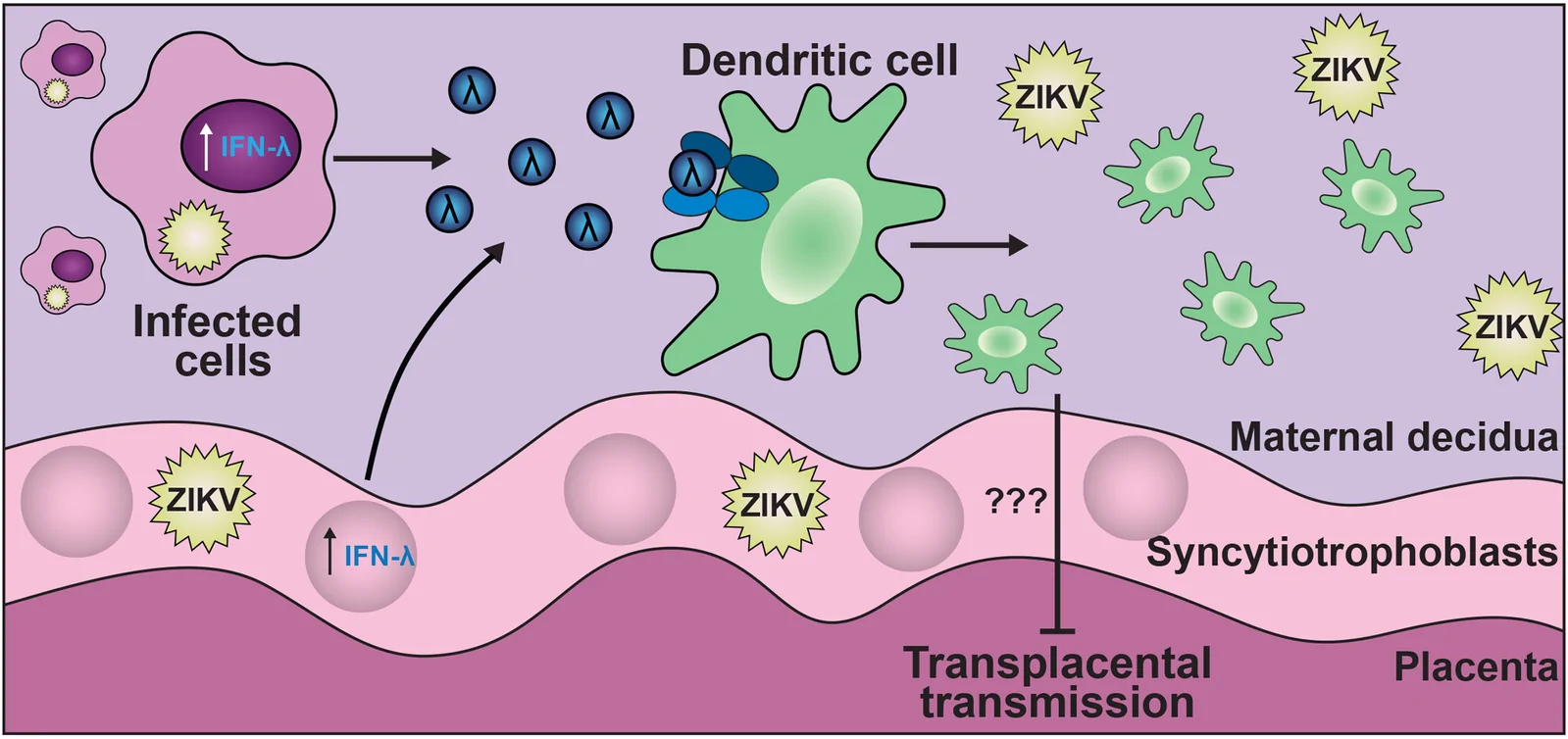
Proposed mechanism by which maternal IFN-λ signaling protects against transplacental transmission during congenital ZIKV infection. Infected cells produce IFN-λ, which signals to maternal dendritic cells. Dendritic cells infiltrate the maternal-fetal interface and block fetal infection through an unknown mechanism.

In dams that lack IFN-λ signaling, we observed increased fetal viral loads and transplacental transmission compared to dams with intact IFN-λ signaling, but these higher fetal viral loads did not correspond to increased fetal pathology. In this study, we used *Ifnar1*^−/−^ dams and sires to achieve more robust ZIKV infection than is sustained in mice treated with an IFNAR1-blocking mAb (*57*), but this mating scheme results in *Ifnar1*^−/−^ fetuses and placentas. Because fetal and/or placental IFN-αβ signaling is a significant driver of fetal pathology and resorptions (*44*, *45*), the relatively low levels of fetal pathology observed in our experiments are not unexpected. Furthermore, in our model, observable fetal pathology is limited to low fetal weights (IUGR) or resorptions, which does not account for placental pathology, more subtle fetal developmental defects, or long-term neurological defects. Congenital ZIKV in mice can result in impaired angiogenesis, neurological defects including brain and spinal cord malformations, and subsequent neurological disorders including paralysis and behavioral changes (*58*–*63*), consistent with outcomes evident in children infected with ZIKV in utero (*3*, *64*–*66*). It is possible that higher fetal viral loads correlate with one or more of these known outcomes of congenital ZIKV infection in mice, even in the absence of IUGR or resorption.

In humans, ZIKV evades the IFN-αβ response by degrading signal transducer and activator of transcription 2 (STAT2), allowing for efficient infection and replication in human cells (*67*–*69*). ZIKV cannot antagonize mouse STAT2 and so does not replicate efficiently in wild-type mice (*57*, *70*, *71*). Therefore, ZIKV pathogenesis is studied in mice lacking the IFN-αβ receptor or in mice with a humanized STAT2 (*57*, *70*). Our model uses mice that lack the IFN-αβ receptor genetically (*Ifnar1^−/−^*) or by treatment with an IFNAR1-blocking mAb, which is sufficient to promote ZIKV infection. Because there is substantial overlap between the IFN-λ and IFN-αβ responses, using an IFNAR1-deficient system allows us to investigate the effects of IFN-λ specifically, distinct from the effects of IFN-αβ. However, in addition to its antiviral effects, the IFN-αβ response also plays an important role in fetal development and placental homeostasis; effects of ZIKV infection on these IFN-mediated processes will not be evident in our experimental system (*72*). Mouse models provide significant advantages for studying immune mechanisms during pregnancy, due to the feasibility of conducting sufficiently powered studies, the availability of transgenic knockout lines to interrogate immune pathways of interest, and the ability to use genetic approaches to differentially modulate maternal versus fetal immune responses. Nonetheless, limitations of mouse pregnancy models include differences in the architecture of mouse placentas compared to human placentas and the shorter duration of mouse pregnancy compared to human pregnancy (∼3 weeks versus ∼40 weeks) (*1*, *17*, *73*). Mouse systems can only address the effects of IFN-λ2 and IFN-λ3 as mice do not produce IFN-λ1 or IFN-λ4. Furthermore, the expression pattern of IFNLR1 may differ in mice compared to humans, potentially leading to differences in the set of IFN-λ–responsive cell types in mice (*74*, *75*).

Because ZIKV is a mosquito-borne virus that causes viremia in humans and animal models, a straightforward mechanism for the virus to spread to the fetal compartment would be hematogenous transplacental transmission, spreading from maternal blood, across the placental interface, into the fetal circulation. However, demonstrating the actual route of fetal infection in humans or animal models is challenging (*76*). In pregnant mouse models, dissected placentas typically retain closely associated decidual tissue, so detection of ZIKV RNA in placental homogenates cannot distinguish true placental infection from infection of the maternal decidua; this is particularly important in experiments using *Ifnar1*^−/−^ dams, which sustain high levels of viral infection. ZIKV infection of the placenta has been detected by immunohistochemistry and RNA hybridization approaches in mice and macaques (*46*, *47*, *61*, *63*), but this could occur secondary to fetal infection by other routes. Histologic analysis of infected mouse placentas often removes this tissue from the uterus before processing, precluding an understanding of how placental infection corresponds to infection of other tissues at the maternal-fetal interface. In this study, we embedded the entire gravid uterus for histologic analysis, preserving the spatial architecture of the fetal compartment within the uterus. This method required pregnancies to be harvested at E13 because, at our standard harvest time of E15, the gravid uterus is too large to fit into a standard tissue cassette and the more extensive fetal development produced poorer-quality tissue sections. Using RNAscope, we detected ZIKV RNA in the uterus, placenta, and fetus, but the most abundant ZIKV RNA signal was detected in the uterus, identifying this tissue as a key site of ZIKV infection at the maternal-fetal interface and consistent with RT-qPCR data showing that the uterus has higher viral loads per gram compared to the placenta or fetus. We only examine one plane of the gravid uterus, allowing for the possibility that ZIKV spatial distribution changes across different planes of tissue. Notably, a study in a rhesus macaque model of congenital ZIKV infection detected robust infection in the uterus and fetal membranes, rather than the placenta, concordant with our results and supporting a model wherein ZIKV spreads to the fetal compartment by a route that bypasses the placenta (*46*, *47*). Such a route would be consistent with findings that the syncytiotrophoblasts that form the interface with maternal blood are highly resistant to infection, including by ZIKV, in part due to constitutive expression of IFN-λ (*40*, *41*, *77*). Our detection of abundant ZIKV RNA signal in the uterus supports the idea that there are additional routes of fetal infection beyond hematogenous transplacental transmission.

We found that *Ifnar1^−/−^ Ifnlr1^−/−^* dams have decreased NK cell and dendritic cell accumulation in the uterus compared to *Ifnar1^−/−^* dams, suggesting that maternal IFN-λ signaling promotes accumulation or infiltration of these cells at the maternal-fetal interface during congenital ZIKV infection. This effect was not perfectly recapitulated in the placenta, where *Ifnar1^−/−^ Ifnlr1^−/−^* dams exhibited a decrease in NK cells and dendritic cells as a percentage of leukocytes but not in the total number of cells. Interpretation of flow cytometry results from dissociated placentas is somewhat confounded by decidual tissue that remains closely associated with the placenta when this tissue is dissected from the gravid uterus, meaning that placental leukocyte preparations likely include decidual leukocytes as well. Because the decidua contains an abundance of leukocytes, particularly decidual NK cells (*78*–*80*), the effect of maternal IFN-λ signaling on uterine NK cells and dendritic cells may contribute to differences observed in the placentas of these mice. Future studies will incorporate congenic markers to more readily distinguish maternal and fetal leukocytes in these mixed-source tissues.

Although we found that maternal IFN-λ signaling corresponded to higher NK cell accumulation in the uterus, when we depleted maternal NK cells we found no effect on fetal infection, arguing against a key role for NK cells in IFN-λ–mediated protection against ZIKV congenital infection. Notably, in other contexts, NK cells have been demonstrated to have important antiviral effects during human cytomegalovirus infection (*81*, *82*). To evaluate the role of NK cells in protection against congenital ZIKV infection, we administered αNK1.1 antibody at the time of infection (E9) and every 2 days until harvest (E15), resulting in a decrease in NK cells in the blood, spleen, uterus, and placentas as assessed by flow cytometry. Notably, although leukocyte depletions by administration of antibodies have been performed in pregnant mice (*83*–*87*) and have been shown to deplete target cells in the pregnant uterus (*88*), to our knowledge, it had not previously been demonstrated that this approach successfully depletes target cells in the placenta; our results demonstrate that this approach can be used to further investigate immune-mediated protection in the placenta, as well as in maternal tissues. Although we observed successful depletion of NK cells at the maternal-fetal interface when tissues were harvested at E15, it remains possible that, at the time of infection or throughout infection, there were sufficient NK cells present at the maternal-fetal interface to contribute to protection against fetal infection. In addition, although NK cells may not have an important role in protection against fetal infection in this model, it is possible that NK cells accumulate at the maternal-fetal interface in a maternal IFN-λ signaling–dependent manner for other purposes. NK cells are critical cell types during pregnancy and play important roles in development of the fetus and placenta including aiding in the formation of the placenta and spiral artery, contributing to immune tolerance and promoting fetal development (*89*, *90*). NK cells may infiltrate the maternal-fetal interface during congenital ZIKV infection to promote immune tolerance in the context of the robust antiviral response promoted by maternal IFN-λ signaling. Furthermore, although NK cells mediate protection in an IFN-λ–dependent manner in other contexts, it remains unclear whether NK cells are IFN-λ responsive (*52*, *91*, *92*), suggesting that other IFN-λ–responsive cells may be involved in recruiting NK cells to the maternal-fetal interface.

Our results show that dendritic cells accumulate at the maternal-fetal interface and protect against ZIKV fetal infection in a manner dependent on maternal IFN-λ signaling, but the mechanism by which dendritic cells mediate protection against fetal infection remains unknown. The IFN-λ–dependent accumulation of dendritic cells at the maternal-fetal interface may promote a more robust antiviral response, perhaps one more optimally localized to specific sites of ZIKV infection. Circulating myeloid cells, including dendritic cells, are key targets for ZIKV infection, with these cells often serving as some of the first cells to become infected in the skin and the genital tract (*93*–*95*). It is possible that dendritic cells at the maternal-fetal interface are infected with ZIKV, but maternal IFN-λ signaling to these cells produces an antiviral response that limits spread of the virus beyond infected cells. Furthermore, IFN-λ is known to alter dendritic cell function and the interaction between dendritic cells and CD8 T cells in the context of influenza A virus and SARS-CoV-2 (severe acute respiratory syndrome coronavirus 2) infections (*96*, *97*). Although we did not observe differences in CD4 or CD8 T cell accumulation in the absence of maternal IFN-λ signaling, it is possible that IFN-λ signaling to dendritic cells could affect the quality of antiviral CD8 T cell responses at the maternal-fetal interface.

CD11c is a well-established marker for dendritic cells in mice but is not entirely specific to dendritic cells as a subset of macrophages and monocytes also can express CD11c (*98*). In our flow cytometry experiments, we tried to mitigate any confounding effects by considering CD11c^hi^ cells as dendritic cells and CD11c^int^ F4/80^+^ cells as macrophages. We and others describe conditional knockout mice using *CD11c*-Cre as having dendritic cell–specific deletions (*99*–*103*). However, it is possible that increased fetal viral loads and transplacental transmission observed in *CD11c*-Cre-*Ifnlr1*^−/−^ dams could be due to lack of IFN-λ signaling in CD11c^+^ monocytes or macrophages, which will be a consideration to examine in future work.

Our group and others have demonstrated that neutrophils are a key IFN-λ–responsive cell type in mice in a variety of infection and inflammatory contexts (*28*, *53*, *75*, *104*, *105*). However, our experiments using conditional knockout mice found that dams lacking IFN-λ signaling in neutrophils (*Mrp8*-Cre-*Ifnlr1*^−/−^) had similar fetal viral loads and transplacental transmission rates as dams with intact IFN-λ signaling, indicating that neutrophils are not key IFN-λ–responsive cells mediating protection of the fetus during congenital ZIKV infection. The effect of maternal IFN-λ signaling on neutrophil accumulation in the uterus and placenta was difficult to assess in our flow cytometry experiments, due to technical obstacles to distinguishing neutrophils in the circulation from neutrophils in tissue; future studies will incorporate in vivo intravascular staining approaches to better assess neutrophil abundance and activity at the maternal-fetal interface.

Together, our data provide the first evidence for regulation of dendritic cell function by IFN-λ signaling at the maternal-fetal interface during congenital infection. Using a combination of spatial analysis and immune phenotyping approaches, we have shown that maternal IFN-λ signaling limits ZIKV transplacental transmission, amplifies IFN-λ expression at the site of infection, and promotes the accumulation of NK cells and dendritic cells at the maternal-fetal interface during congenital ZIKV infection. Human placentas and uterine tissue respond similarly to IFN-λ as mouse placentas and uterine tissue, suggesting broader implications for our work (*41*, *42*, *44*, *54*). Our results further highlight the importance of the maternal immune response during congenital infections.

## MATERIALS AND METHODS

### Cells and viruses

Vero (African green monkey kidney epithelial) cells were maintained in Dulbecco’s modified Eagle’s medium (DMEM) containing 5% heat-inactivated fetal bovine serum (FBS) and 1X GlutaMAX (Gibco, #35050061) at 37°C with 5% CO_2_. Virus stocks were grown in Vero cells in DMEM containing 2% FBS and 10 mM Hepes at 37°C with 5% CO_2_. ZIKV strain FSS13025 (Cambodia 2010) was obtained from the World Reference Center for Emerging Viruses and Arboviruses (*106*). Virus stock titers were determined by focus-forming assay using anti-flavivirus human mAb E60 (*107*) at 100 ng/ml (purified from hybridoma supernatant by the UNC Protein Expression Core Facility), horseradish peroxidase–conjugated goat anti-mouse antibody at a 1:5000 dilution (Fisher, #50-673-91), and TrueBlue Peroxidase substrate (KPL). Antibody incubations were performed for 2 hours at room temperature. Foci were counted on a CTL Immunospot analyzer.

### Mice

All experiments and husbandry protocols were approved by the Institutional Animal Care and Use Committee at the University of North Carolina at Chapel Hill (protocol nos. 25-132 and 22-149). Experiments used 8- to 20-week-old female mice on a C57BL/6 background mated with 8- to 52-week-old male mice on a C57BL/6 background. *Ifnar1^−/−^* mice and *Ifnar1^−/−^ Ifnlr1^−/−^* mice were bred in-house as knockout x knockout (*44*). *Actin-*Cre-*Ifnlr1^−/−^*, *LysM-*Cre-*Ifnlr1^−/−^*, *Mrp8-*Cre-*Ifnlr1^−/−^*, and *CD11c-*Cre-*Ifnlr1^−/−^* mice were generated by crossing *Ifnlr1^f/f^* mice with mice expressing Cre recombinase under control of the *Actin* promoter (Jackson Laboratory, #19099; obtained from J. Ting, UNC Chapel Hill), *LysM* promoter (Jackson Laboratory, #4781; obtained from J. Ting, UNC Chapel Hill), *Mrp8* promoter (Jackson Laboratory, #21614), or *Cd11c* promoter (Jackson Laboratory, #8068; obtained from J. Ting, UNC Chapel Hill) (*53*). Mice were bred as Cre hemizygotes, generating mixed litters in which 50% of mice were Cre+, lacking IFN-λ signaling in respective cell types, and 50% of mice were Cre−, retaining IFN-λ signaling. Experiments were performed blinded to Cre status, with tail snips from experimental mice being genotyped after data collection by PCR for both Cre and *Ifnlr1*.

### Genotyping

For conditional knockout mice, *Ifnlr* and Cre genotypes were determined by PCR on tail samples following harvest of pregnant dams. DNA was extracted from tails using the Quantabio DNA extraction kit (#95091-250). PCR was run using the Accustart II Geltrack PCR Supermix (#89235-014) and the following primers: *Ifnlr*: F^1^ 5′-AGGGAAGCCAAGGGGATGGC-3′, R^1^ 5′-AGTGCCTGCTGAGGACCAGGA-3′, and R^3^ 5′-GGCTCTGGACCTACGCGCTG-3′; *Actin*-Cre: F^1^ 5′-CGTACTGACGGTGGGAGAAT-3′ and R^1^ 5′-CCCGGCAAAACAGGTAGTTA-3′; *Cd11c*-Cre: F^1^ 5′-ACTTGGCAGCTGTCTCCAAG-3′ and R^1^ 5′-GCGAACATCTTCAGGTTCTG-3′; *LysM*-Cre: F^1^ 5′-CTTGGGCTGCCAGAATTTCTC-3′, R^1^ 5′-CCCAGAAATGCCAGATTACG-3′, and R^2^ 5′-TTACAGTCGGCCAGGCTGAC-3′; and *Mrp8*-Cre: F^1^ 5′-GCGGTCTGGCAGTAAAAACTATC-3′ and R^1^ 5′-GTGAAACAGCATTGCTGTCACTT-3′.

For *Actin*-Cre, *Cd11c*-Cre, and *Mrp8*-Cre, genotyping, two primers (F^1^ 5′-CAAATGTTGCTTGTCTGGTG-3′ and R^1^ 5′-GTCAGTCGAGTGCACAGTTT-3′) were used to amplify an internal positive control.

### Mouse experiments

Females were exposed to soiled male bedding 3 days before mating to promote estrus. Male and female pairs were cohoused overnight (E0) and were separated in the morning (E1). Female mice were inoculated via subcutaneous injection in the footpad with 1000 FFU of ZIKV in 50 μl 9 days postmating (E9). Conditional knockout mice were administered 2 mg of anti–IFNAR1-blocking antibody MAR1-5A3 via intraperitoneal injection 1 day before infection (E8) (*57*). Pregnant mice were harvested 6 dpi (E15) or 8 dpi (E17). Cardiac puncture was used to harvest maternal blood in serum separator tubes (BD). Serum was separated from whole blood by centrifugation at 8000 rpm for 5 min. Fetuses, placentas, and maternal spleens were harvested and weighed. Photographs of fetuses and placentas were taken at time of harvest. Any implantation without discrete fetal and placental structures was considered a resorption. For microscopy experiments, pregnant mice were harvested 4 dpi (E13). Dams were perfused with 20 ml of phosphate-buffered saline (PBS), and then the entire gravid uterus was harvested, or fetuses, placentas, and maternal uteruses were harvested and weighed.

### Viral genome quantification by RT-qPCR

Tissues were homogenized in 1 ml of PBS using a MagNA Lyser (Roche), and equal parts homogenate was added to equal parts RLT buffer (Qiagen) (150 μl for fetuses and placentas and 300 μl for spleens and uteruses). Viral RNA was extracted using a Qiagen RNeasy kit and detected by TaqMan one-step RT-qPCR using the following primer/probe set: forward-TTGGTCATGATACTGCTGATTGC; reverse-CCTTCCACAAAGTCCCTATTGC; and probe56-FAM/CGGCATACA/ZEN/GCATCAGGTGCATAGGAG/3IABkFQ on a Bio-Rad CFX96. ZIKV copies per milliliter were determined by comparison to a standard curve of 10-fold dilutions of the ZIKV-A plasmid (*108*).

### Plaque assay

Tissues were homogenized in 1 ml of PBS and silicone beads using a MagNA Lyser (Roche) and serially diluted in a round-bottom 96-well plate. Beginning with the undiluted sample, 100 μl of each dilution was added to monolayers of Vero cells (6-well plate with 5 × 10^5^ cells per well plated 1 day prior) with 500 μl of DMEM with 2% FBS and 10 mM HEPES, andincubated for 1 hour at 37°C. After 1 hour, media was removed and 2 ml of methylcellulose (1% methylcellulose in 1X MEM and 2% FBS, l-glutamine, P/S, and HEPES) was added to the wells and left to incubate at 37°C for 5 days. After 5 days, media was aspirated, and the plates were fixed by adding 500 μl of 10% formaldehyde for at least 30 min at room temperature. After fixing, paraformaldehyde (PFA) was poured off, plates were washed with tap water, stained with crystal violet (1% crystal violet, 20% ethanol, and 80% water), and washed again with tap water. Plaques were counted after plates dried at room temperature overnight.

### HiPlex RNAscope

The entire gravid uterus was harvested and fixed in 10% neutral buffered formalin for 72 hours. Tissues were processed using a Sakura Tissue-Tek VIP 5 Tissue Processor, embedded in paraffin, and sectioned into 5-μm slices. In situ hybridization was performed using ACDBio’s RNAscope technology per the kit instructions [RNAscope Kit HiPlex12 Detection kit V2 (488, 550, 650, 750), #324400]. Tissue sections were deparaffinized and hydrated, followed by antigen retrieval for 15 min at 100°C. Tissue sections were incubated with protease for 30 min at 40°C. Then, probes were hybridized for 2 hours at 40°C. Following hybridization, probe signal was amplified. The following probes were used: V-ZIKV-T1 (#467771), Mm-Ifnl2-T2 (#410231), Mm-Ifnb1-T3 (#406531), Mm-Ifng-T4 (#311391), Mm-Ifit1-T5 (#500071), Mm-ITGAM-T6 (#311491), Mm-ITGAX-T7 (#311501), Mm-Cxcl9-T8 (#489341), Mm-Tpbg-T9 (#425681), Mm-Ifnlr1-T10 (#512981), Mm-Cd244-T11 (#486801), and Mm-Ngp-T12 (#1041701). Fluorophores for T1 to T4 were developed in the first round, and then coverslips were added to slides using Fluorogel II with 4′,6-diamidino-2-phenylindole (DAPI; Electron Microscopy Sciences, #17985-50). Tissue sections were imaged, and then according to kit instructions, coverslips were removed, fluorophores for T1 to T4 were cleaved, fluorophores for T5 to T8 were developed, and coverslips were added. Tissue sections were imaged, and the coverslip removal, fluorophore cleavage, and subsequent development process was repeated for T9 to T12. Following imaging of T9 to T12, fluorophores were cleaved, coverslips were added to slides, and sections were imaged without any fluorophores as a control for background fluorescence.

### Microscopy and image analysis

The samples were imaged following each round of probe hybridization using a using a Nikon Eclipse Ti2-D-LHLED (Nikon, #MEE55700) with a linear encoded mechanical stage (Nikon, #MEC56130). All images were taken with an air immersion 4x Plan Apo objective with a numerical aperture of 0.2 (Nikon, #MRD00045). The following filters and exposure times were used for detection of fluorescence signal: green fluorescent protein (GFP; Chroma, 49002-ET-GFP, exposure time of 400 ms), TexasRed (Chroma, 49008-ET-mCherry, Texas Red, exposure time of 600 ms), Narrow Cy5 (Chroma, 49009-ET-Cy5 Narrow Excitation, exposure time of 400 ms), DAPI (Chroma, NC764537-49000-ET- DAPI, exposure time of 400 ms), and Cy7 (Chroma, 96367-ET-Cy7, exposure time of 400 ms). All filter cubes were hard coated. Filter cubes were in a motorized filter cube turret (Nikon Ti2-F-FLT-E, #MEV51030). Images were detected with a pco.edge 4.2 Q High sCMOS camera (Pco #77067008). This camera has a 16-bit maximum bit depth. NIS-Elements was the acquisition software. Images were collected across the entire tissue section on the slide, stitched in NIS-Elements (version AR), denoised using the Nikon Batch Denoiser, and subsequently converted to Imaris files. Images of each round of hybridization on the same sample were registered using the SITK-IBEX–based registration package in Python (*109*). Using Imaris (version 10.2.0) spot detector, spots were assigned to the signal from probes based on the quality of the signal with the following parameters: ZIKV, 170.0; *Ifnl2/3*, 163.0; and *Itgax*, 25.0. The shortest distance between spots of interest was calculated in Imaris. Images shown are representative.

### Cellular gene expression measured by RT-qPCR

Gene expression was measured by extracting RNA from placental tissue as previously described, followed by cDNA synthesis with the Verso cDNA Synthesis Kit (Thermo Fisher Scientific). Reactions were set up according to the manufacturer’s instructions. Quantification of gene expression was performed using the Power SYBR Green PCR Master Mix (Applied Biosystems) using the following primer combinations on a Bio-Rad CFX96: *Actb* (Fwd-CCTCTATGCCAACACAGTGC; Rev-CCTGCTTGCTGATCCACATC), *Ifnl3* (Fwd-GACAAGAACCCAAGCTGACC; Rev-ATGTCCTTCTCAAGCAGCCT), *Ifnb1* (Fwd-GTCCTCAACTGCTCTCCACT; Rev-CCTGCAACCACCACTCATTC), and *Ifit1* (Fwd-GATGGACTGTGAGGAAGGCT; Rev-TGGATTTAACCGGACAGCCT).

### Flow cytometry

Tissues were harvested into cold PBS and processed using established methods for digestion of mouse uteruses (*110*) and a modified method for digestion of mouse placentas. Gravid uteruses were harvested at E15 and dissected to separate the uterus, fetuses, and placentas. Uteruses were cut into ∼0.5-cm pieces, and excess fat was trimmed and discarded. Then, uteruses were incubated in a predigestion solution (Hanks’ balanced salt solution with 5% FBS, 5 mM EDTA, and 1 mM dithiothreitol) for two 30-min periods at 37°C. Intraepithelial leukocytes were collected after each period by passing uteruses through 70-μm cell strainers. Intraepithelial leukocytes and uterus samples were combined and then dissociated using a GentleMACS (Miltenyi, #130-134-029) and enzymes from the mouse lamina propria dissociation kit (Miltenyi, #130-097-410). Uterus samples were passed through 70- and 40-μm filters, centrifuged at 350*g* for 5 min, and resuspended in PBS with 1% FBS for staining with fluorescent antibodies. Separately, placentas were dissected into eight pieces and washed with PBS. Placentas were then dissociated using a GentleMACS and enzymes from the mouse lung dissociation kit (Miltenyi, #130-095-927). Samples were incubated at 37°C for 30 min and then dissociated again using the GentleMACS. Red blood cells (RBCs) were lysed using RBC lysis buffer (155 mM NH_4_Cl, 12 mM NaHCO_3_, and 0.1 mM EDTA), washed with PBS, and centrifuged at 500*g* for 5 min. Placental samples were passed through a 70-μm cell strainer, centrifuged at 300*g* for 10 min, and resuspended in PBS with 1% FBS (staining buffer), counted, and stained with fluorescent antibodies.

Spleens and whole blood were collected to confirm depletion of NK cells. Spleens were harvested into cold DMEM (Gibco) containing DNase I (100 μg/ml; Fisher, #NC9185812) and Collagenase P (1 mg/ml; Sigma-Aldrich, #11249002001) and then passed through 70-μm filters and suspended in DMEM and 5% FBS. RBCs were lysed using ACK lysis buffer (Gibco, #A1049201), and samples were washed with PBS, centrifuged at 300*g* for 5 min, and resuspended in staining buffer and stained with fluorescent antibodies. Whole blood was collected via cardiac puncture into tubes containing EDTA (Fisher, #02-669-33). RBCs were lysed with ACK lysis buffer, centrifuged at 500*g* for 5 min, and resuspended in staining buffer for counting and staining with fluorescent antibodies.

Fc Block and all fluorescent antibodies used were sourced from BioLegend and titrated before experimental use. All samples were treated with Fc Block on ice for 10 min. Samples were washed with staining buffer and stained for surface antigens on ice for 30 min, washed three times with staining buffer, and fixed with 2% PFA. Samples were analyzed on an Attune Nxt (Thermo Fisher Scientific), and data were analyzed with the FlowJo Software (v10.10). The following antibodies were used: αCD3 (17A2), αCD4 (GK1.5), αCD8 (53-6.7), αCD11b (M1/70), αCD11c (N418), αCD19 (6D5), αCD45 (30-F11), αF4/80 (BM8), αLy6C (HK1.4), αLy6G (1A8), αMHCII (M5/114.15.2), αNK1.1 (PK136), αTCRγδ (GL3), and Zombie Aqua Fixable Dye. Gating strategies for NK cell and dendritic cells are shown in fig. S6. All gating strategies were based on published immunophenotyping methods (*111*–*113*). Fluorescence minus one controls and single-color controls were used for gating and compensation.

### NK cell depletion

To deplete NK cells, mice were injected intraperitoneally on E9, E11, and E13 with 100 μg of αNK1.1 antibody (clone PK136, BioXCell). Depletion of NK cells in blood, spleen, uterus, and placentas was confirmed by flow cytometry at the end of the experiment in each cohort of mice.

### Statistics

All statistics were calculated using GraphPad Prism 10 or R. Viral loads were analyzed using a Mann-Whitney *U* test. Transplacental transmission and fetal pathology rates were analyzed using a Cochran-Armitage test. Flow cytometry data were analyzed using an unpaired *t* test.
