# Supplementary material for: Interferon-λ signaling to maternal dendritic cells protects against congenital Zika virus infection
Source: Sci Adv. 2026 Oct 23;12(43):eaeh1073. doi: 10.1126/sciadv.aeh1073 (PMC13693664; doi:10.1126/sciadv.aeh1073)
Supplement: Supplementary file 1 [file sciadv.aeh1073_sm.pdf]

Science Advances
AAAS

Supplementary Materials for

Interferon-λ signaling to maternal dendritic cells protects against congenital Zika virus infection

Margaret R. Dedloff *et al.*

Corresponding author: Helen M. Lazear, helen.lazear@med.unc.edu

Sci. Adv. **12**, eaeh1073 (2026)
DOI: 10.1126/sciadv.aeh1073

The PDF file includes:

Figs. S1 to S7
Legends for tables S1 and S2

Other Supplementary Material for this manuscript includes the following:

Tables S1 and S2

Supplementary figure legends

Figure S1. Maternal IFN-λ signaling results in decreased infectious viral titers in fetuses. Dams from *Ifnar1*$^{-/-}$ x *Ifnar1*$^{-/-}$ *Ifnlr1*$^{-/-}$ or *Ifnar1*$^{-/-}$ *Ifnlr1*$^{-/-}$ x *Ifnar1*$^{-/-}$ crosses were inoculated at day 9 post-mating (E9) with ZIKV FSS13025 by subcutaneous injection in the footpad. Fetal tissues were harvested at E15. (A) Viral loads in the fetus were measured by plaque assay. Each data point represents one fetus. (B) Transplacental transmission was calculated as percent of fetuses with viral loads above the limit of detection. Results are combined from 62 fetuses from 9 *Ifnar1*$^{-/-}$ dams and 82 fetuses from 10 *Ifnar1*$^{-/-}$ *Ifnlr1*$^{-/-}$ dams per group from 11 independent experiments. Viral loads were compared by Mann-Whitney test, proportions by Cochran Armitage test (*, $P < 0.05$).

Figure S2. IFN-λ protects against fetal infection regardless of infection duration. (A) Dams from *Ifnar1*$^{-/-}$ x *Ifnar1*$^{-/-}$ *Ifnlr1*$^{-/-}$ or *Ifnar1*$^{-/-}$ *Ifnlr1*$^{-/-}$ x *Ifnar1*$^{-/-}$ crosses were inoculated at day 9 post-mating (E9) with ZIKV FSS13025 by subcutaneous injection in the footpad. Fetal tissues were harvested at E15 or E17. (B) ZIKV viral loads in the fetus were measured by RT-qPCR. Each data point represents one fetus. (C) Transplacental transmission was calculated as percent of fetuses with viral loads above the limit of detection. (D) Proportions of fetuses exhibiting resorption. Results are combined from 91 fetuses from 10 *Ifnar1*$^{-/-}$ dams and 66 fetuses from 8 *Ifnar1*$^{-/-}$ *Ifnlr1*$^{-/-}$ dams (E15 harvest) and 99 fetuses from 12 *Ifnar1*$^{-/-}$ dams and 58 fetuses from 7 *Ifnar1*$^{-/-}$ *Ifnlr1*$^{-/-}$ dams (E17). Viral loads were compared by Mann-Whitney test and proportions were compared by Cochran Armitage test (****, $P < 0.0001$; **, $P < 0.01$; *, $P < 0.05$).

Figure S3. Spatial analysis of the maternal-fetal interface during congenital ZIKV infection. (A) Sections of gravid uteruses. Dams from *Ifnar1*$^{-/-}$ x *Ifnar1*$^{-/-}$ *Ifnlr1*$^{-/-}$ or *Ifnar1*$^{-/-}$ *Ifnlr1*$^{-/-}$ x *Ifnar1*$^{-/-}$ crosses were inoculated at E9 with ZIKV FSS13025 by subcutaneous injection in the footpad. Gravid uteruses were harvested at E13. Sections of entire gravid uteruses (2 *Ifnar1*$^{-/-}$ dams and 2 *Ifnar1*$^{-/-}$ *Ifnlr1*$^{-/-}$ infected dams, 1 uninfected *Ifnar1*$^{-/-}$ dam, collected in independent experiments; experiment 2 depicted in Figure 2B) were probed for ZIKV RNA (green) and stained with DAPI (blue). (B) Distance of *Ifnl2/3* spots to nearest ZIKV spots was quantified for experiment 2 using the image analysis pipeline in Figure 3A. Each data point represents one *Ifnl2/3* spot. Spots greater than 500μm to the nearest ZIKV spot are not graphed (580 spots not graphed out of 23,777 total spots from *Ifnar1*$^{-/-}$ dam; 1,189 spots not graphed out of 2,937 total spots from *Ifnar1*$^{-/-}$ *Ifnlr1*$^{-/-}$ dam). Black horizontal line indicates the median. Distances were compared by Mann-Whitney test (****, $P < 0.0001$). (C) *Itgax* spots were quantified from experiment 2.

Figure S4. Immunophenotyping of placentas during ZIKV infection. Dams from *Ifnar1*$^{-/-}$ x *Ifnar1*$^{-/-}$ *Ifnlr1*$^{-/-}$ or *Ifnar1*$^{-/-}$ *Ifnlr1*$^{-/-}$ x *Ifnar1*$^{-/-}$ crosses were inoculated at day 9 post-mating (E9) with ZIKV FSS13025 by subcutaneous injection in the footpad. Placentas were harvested at E15 and analyzed by flow cytometry (38 placentas from 5 *Ifnar1*$^{-/-}$ dams and 59 placentas from 8 *Ifnar1*$^{-/-}$ *Ifnlr1*$^{-/-}$ dams collected in 6 independent experiments). (A) Frequency of CD4 T cells (CD3^{+}CD4^{+} out of CD45^{+}). (B) Frequency of CD8 T cells (CD3^{+}CD8^{+} out of CD45^{+}). (C) Frequency of γδ T cells (CD3^{+}TCRγδ^{+} out of CD45^{+}). (D) Frequency of B cells (CD19^{+} out of CD45^{+}). (E) Frequency of macrophages (CD11c^{int}F4/80^{+} out of CD45^{+}). (F) Frequency of monocytes (CD11b^{+}Ly6C^{+}Ly6G^{-} out of CD45^{+}). (G) Number of each leukocyte type quantified in placentas from *Ifnar1*$^{-/-}$ and *Ifnar1*$^{-/-}$ *Ifnlr1*$^{-/-}$ dams. Groups were compared by unpaired *t*-test (****, $P < 0.0001$; **, $P < 0.01$; *, $P < 0.05$).

Figure S5. Immunophenotyping of the uterus during ZIKV infection. Dams from *Ifnar1*$^{-/-}$ x *Ifnar1*$^{-/-}$ *Ifnlr1*$^{-/-}$ or *Ifnar1*$^{-/-}$ *Ifnlr1*$^{-/-}$ x *Ifnar1*$^{-/-}$ crosses were inoculated at day 9 post-mating (E9) with ZIKV FSS13025 by subcutaneous injection in the footpad. Uteruses were harvested at E15 and analyzed by flow cytometry (4 *Ifnar1*$^{-/-}$ dams 5 *Ifnar1*$^{-/-}$ *Ifnlr1*$^{-/-}$ dams collected in 4 independent experiments). (A) Frequency of CD4 T cells (CD3^{+}CD4^{+} out of CD45^{+}). (B) Frequency of CD8 T cells (CD3^{+}CD8^{+} out of CD45^{+}). (C) Frequency of γδ T cells (CD3^{+}TCRγδ^{+} out of CD45^{+}). (D) Frequency of B cells (CD19^{+} out of CD45^{+}). (E) Frequency of macrophages (CD11c^{int}F4/80^{+} out of CD45^{+}). (F) Frequency of monocytes (CD11b^{+}Ly6C^{+}Ly6G^{-} out of CD45^{+}). (G) Number of each leukocyte type quantified in uteruses from *Ifnar1*$^{-/-}$ and *Ifnar1*$^{-/-}$ *Ifnlr1*$^{-/-}$ dams. Groups were compared by unpaired *t*-test (*, $P < 0.05$).

Figure S6. Representative gating strategy to define NK and dendritic cell populations. Gating strategy to define NK and dendritic cells in placentas/uteruses.

Figure S7. NK cells are not required for IFN-λ mediated protection against transplacental transmission. (A) Dams from *Ifnar1*$^{-/-}$ x *Ifnar1*$^{-/-}$ *Ifnlr1*$^{-/-}$ crosses were inoculated at day 9 post-mating (E9) with ZIKV FSS13025 by subcutaneous injection in the footpad, and treated intraperitoneally at E9, E11, and E13 with 100μg of αNK1.1 antibody. (B-F) Blood (3 untreated dams, 4 treated dams, collected in 4 independent experiments), spleens (4 untreated dams, 4 treated dams, collected in 4 independent experiments), uteruses (4 untreated dams, 4 treated dams, collected in 2 independent experiments), and placentas (38 placentas from 5 untreated

dams, 33 placentas from 6 treated dams, collected in 6 independent experiments) were collected at E15 and analyzed for NK1.1^{+} cells by flow cytometry. Groups were compared by unpaired *t*-test (*, $P < 0.05$; **, $P < 0.01$; ***, $P < 0.001$; ****, $P < 0.0001$). (G) Fetuses (37 fetuses from 4 untreated dams, 33 fetuses from 4 treated dams, collected in 6 independent experiments) were collected at E15 for viral load analysis by RT-qPCR. Each data point represents one fetus. Groups were compared by Mann-Whitney and were found to have no significant difference in viral loads.

Supplementary Data Table 1: Fetal infections. Each dam (*Ifnar1*$^{-/-}$ and *Ifnar1*$^{-/-}$ *Ifnlr1*$^{-/-}$) is listed from viral load experiments in Figure 1, Figure 2, Figure 6, and Supplementary Figure 2, along with the number of fetuses in each pregnancy and the number of fetuses positive for ZIKV by RT-qPCR.

Supplementary Data Table 2: Microscopy quantification. Imaris microscopy analysis software was used to assign spots to fluorescent probe signal, count spots, and to measure distances between spots from experiments presented in Figure 3 and Figure 5.

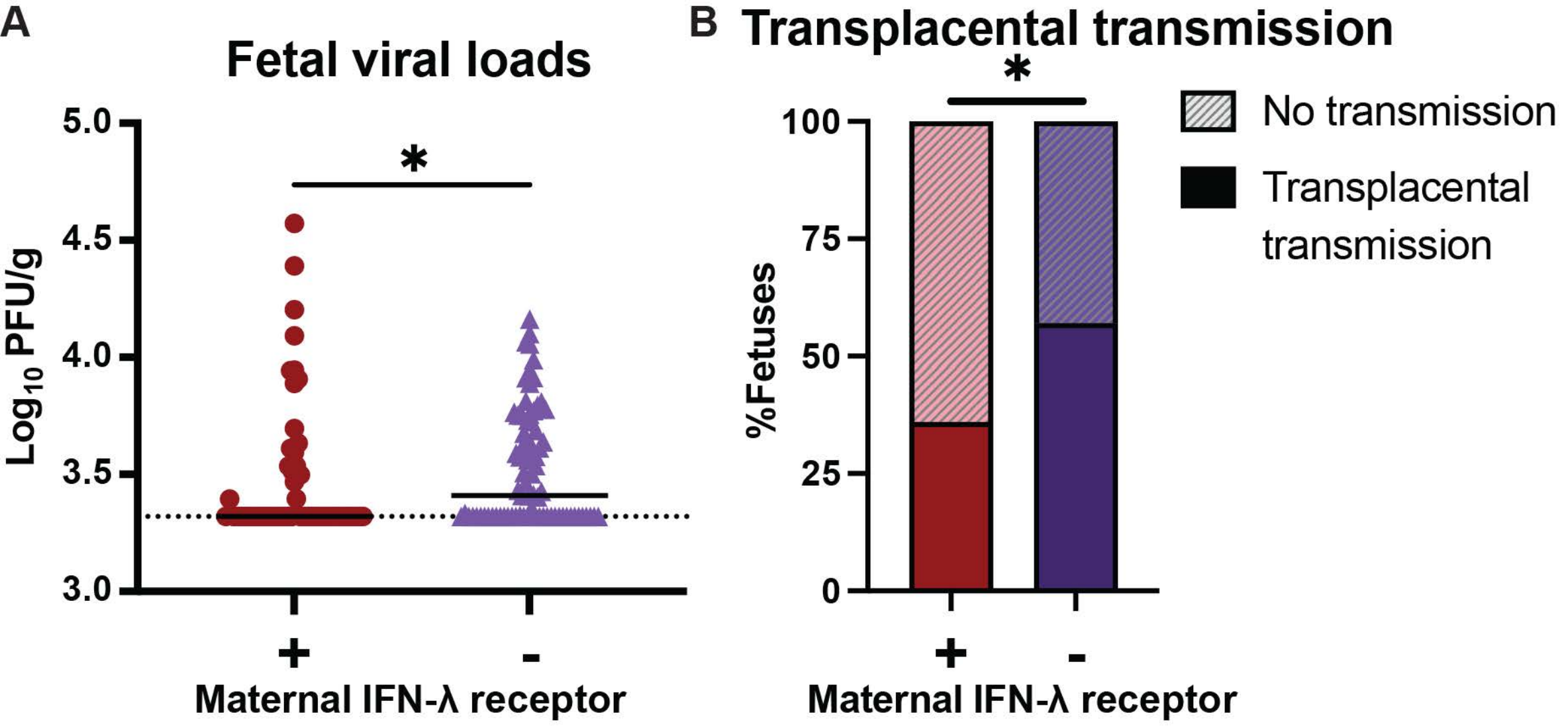
A
Fetal viral loads
5.0
4.5
4.0
3.5
3.0
Log10 PFU/g
*
+
-
Maternal IFN-λ receptor
B
Transplacental transmission
*
100
75
50
25
0
%Fetuses
No transmission
Transplacental transmission
+
-
Maternal IFN-λ receptor

Figure S1

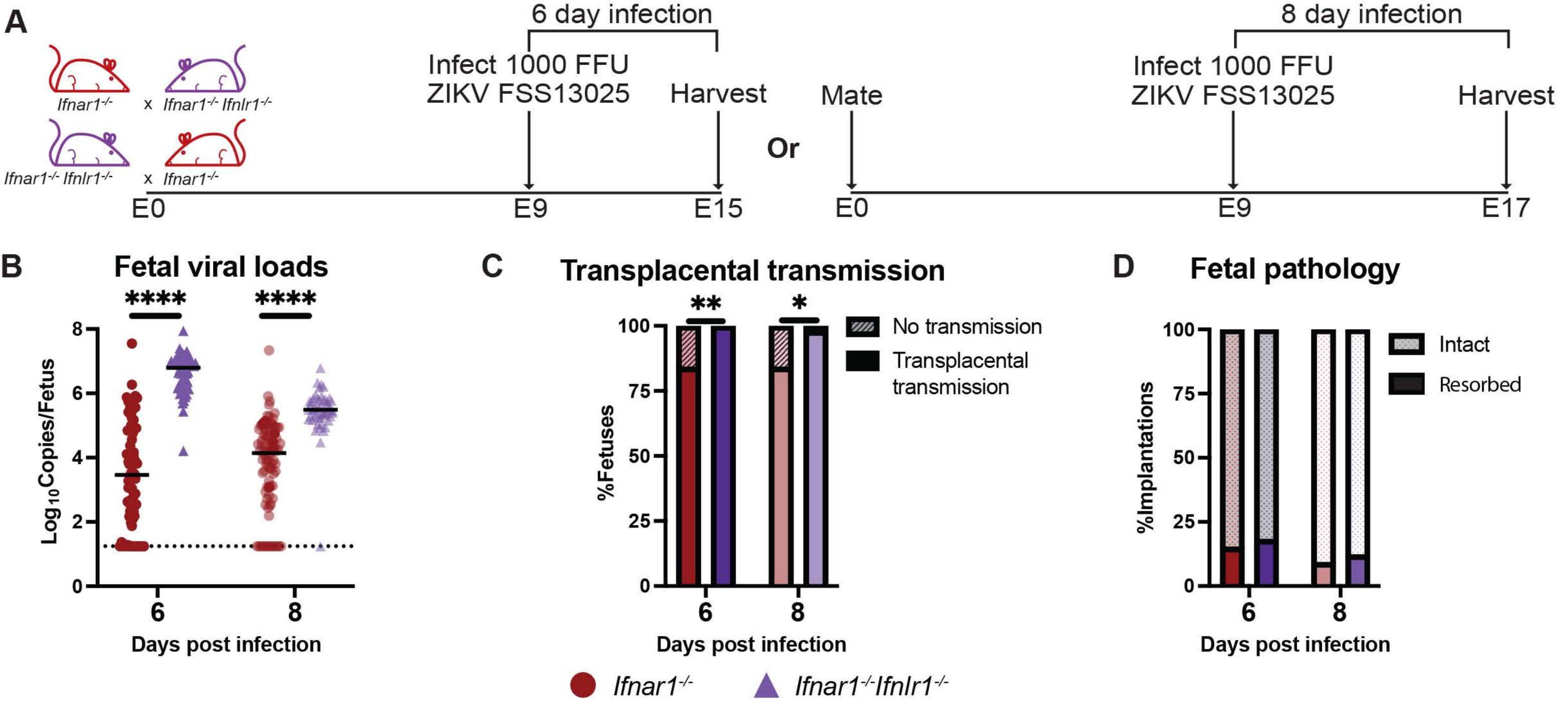
A
Ifnar1-/-
x
Ifnar1-/- Ifnlr1-/-
Ifnar1-/- Ifnlr1-/-
x
Ifnar1-/-
6 day infection
Infect 1000 FFU
ZIKV FSS13025
Harvest
E0
E9
E15
Or
Mate
8 day infection
Infect 1000 FFU
ZIKV FSS13025
Harvest
E0
E9
E17
B
Fetal viral loads
****
****
Log10Copies/Fetus
Days post infection
C
Transplacental transmission
**
*
%Fetuses
No transmission
Transplacental transmission
Days post infection
D
Fetal pathology
%Implantations
Intact
Resorbed
Days post infection
Ifnar1-/-
Ifnar1-/-Ifnlr1-/-

Figure S2

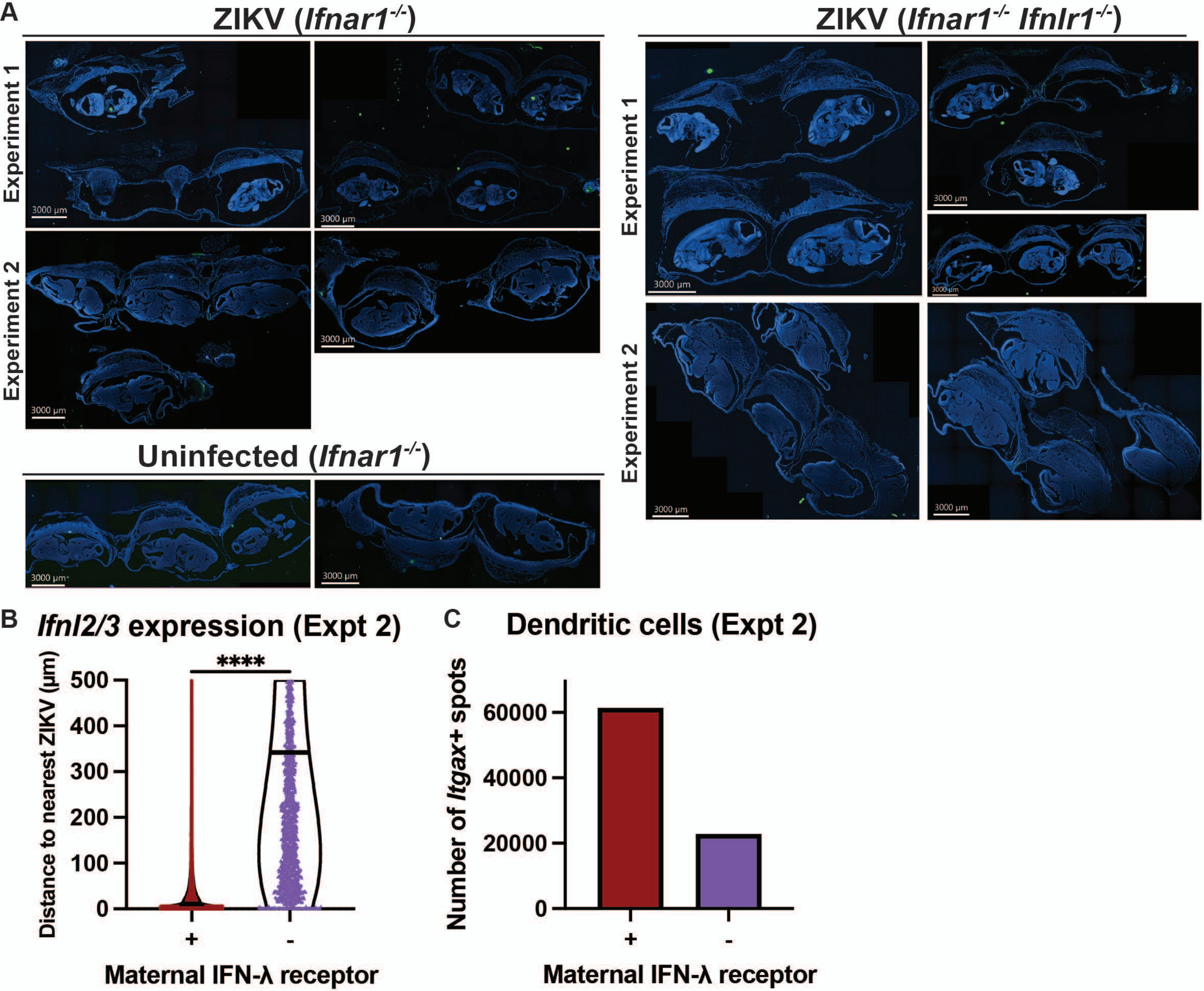

A
ZIKV (Ifnar1-/-)
ZIKV (Ifnar1-/- Ifnlr1-/-)
Experiment 1
Experiment 2
3000 µm
Uninfected (Ifnar1-/-)
B
Ifnl2/3 expression (Expt 2)
****
Distance to nearest ZIKV (µm)
500
400
300
200
100
0
+
-
Maternal IFN-λ receptor
C
Dendritic cells (Expt 2)
Number of Itgax+ spots
60000
40000
20000
0
+
-
Maternal IFN-λ receptor

Figure S3

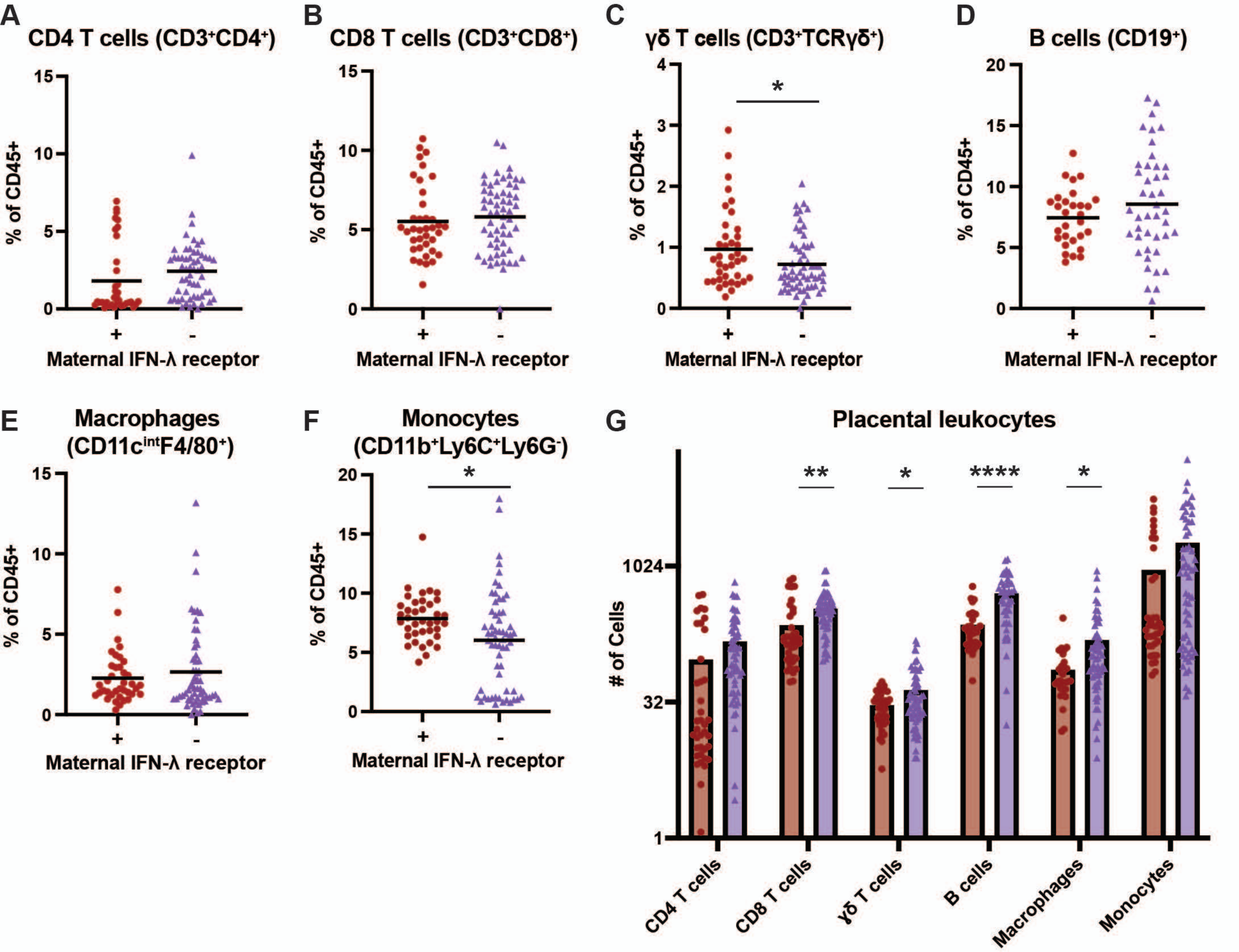
A
CD4 T cells (CD3⁺CD4⁺)
B
CD8 T cells (CD3⁺CD8⁺)
C
γδ T cells (CD3⁺TCRγδ⁺)
D
B cells (CD19⁺)
E
Macrophages (CD11c^int F4/80⁺)
F
Monocytes (CD11b⁺Ly6C⁺Ly6G⁻)
G
Placental leukocytes
% of CD45+
Maternal IFN-λ receptor
+
-
# of Cells
1024
32
1
CD4 T cells
CD8 T cells
γδ T cells
B cells
Macrophages
Monocytes

Figure S4

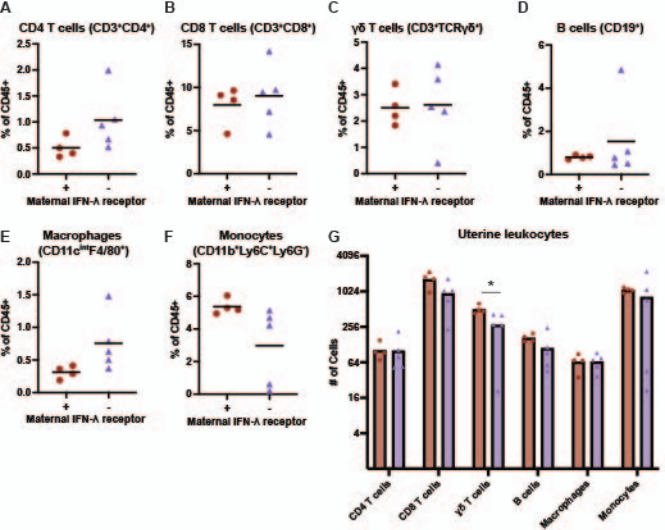
A
CD4 T cells (CD3+CD4+)
B
CD8 T cells (CD3+CD8+)
C
γδ T cells (CD3+TCRγδ+)
D
B cells (CD19+)
% of CD45+
Maternal IFN-λ receptor
E
Macrophages (CD11c^int F4/80+)
F
Monocytes (CD11b+Ly6C+Ly6G-)
G
Uterine leukocytes
# of Cells
4096
1024
256
64
16
4
CD4 T cells
CD8 T cells
γδ T cells
B cells
Macrophages
Monocytes
*

Figure S5

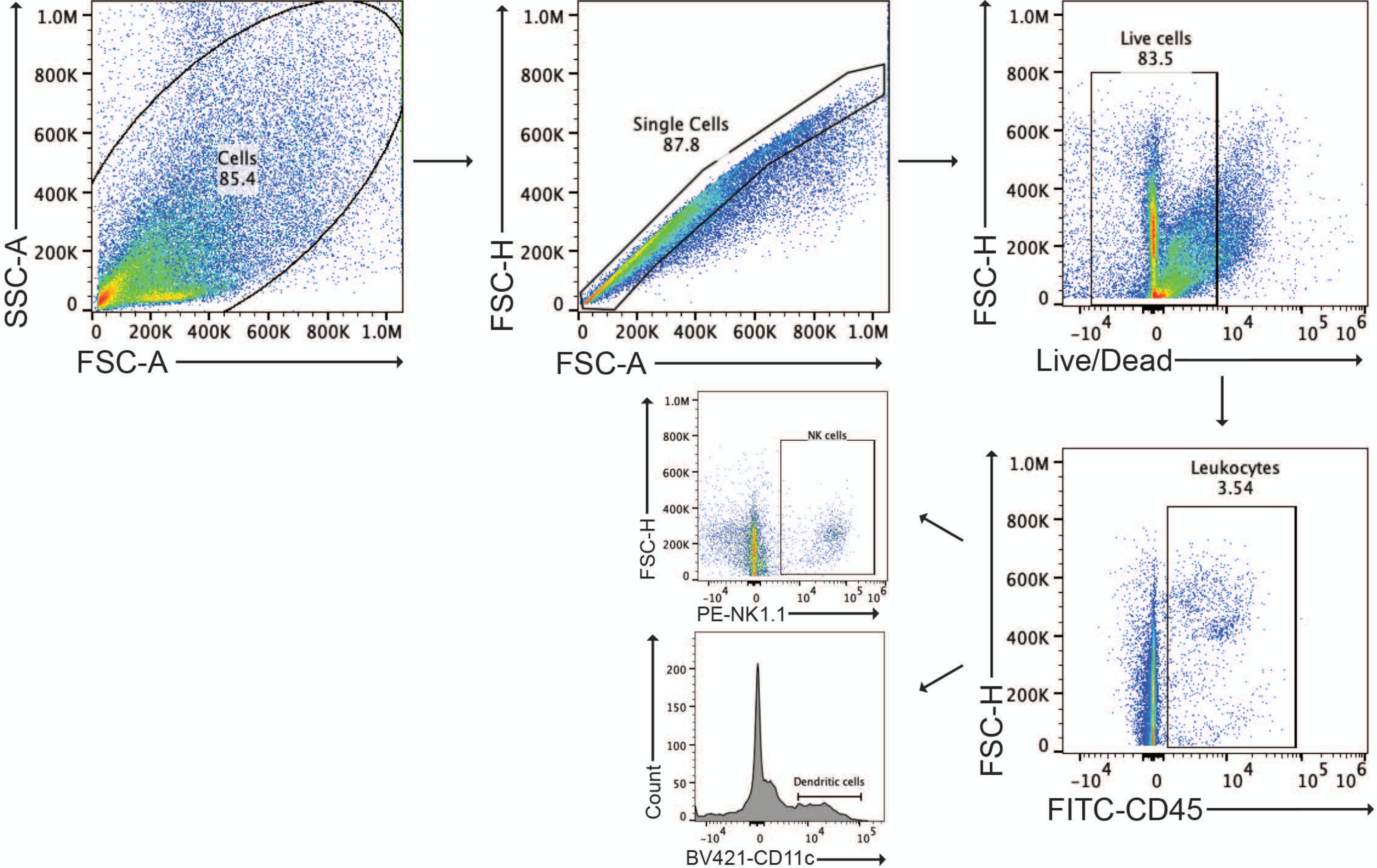

Cells
85.4
SSC-A
FSC-A
Single Cells
87.8
FSC-H
FSC-A
Live cells
83.5
FSC-H
Live/Dead
Leukocytes
3.54
FSC-H
FITC-CD45
NK cells
FSC-H
PE-NK1.1
Dendritic cells
Count
BV421-CD11c

Figure S6

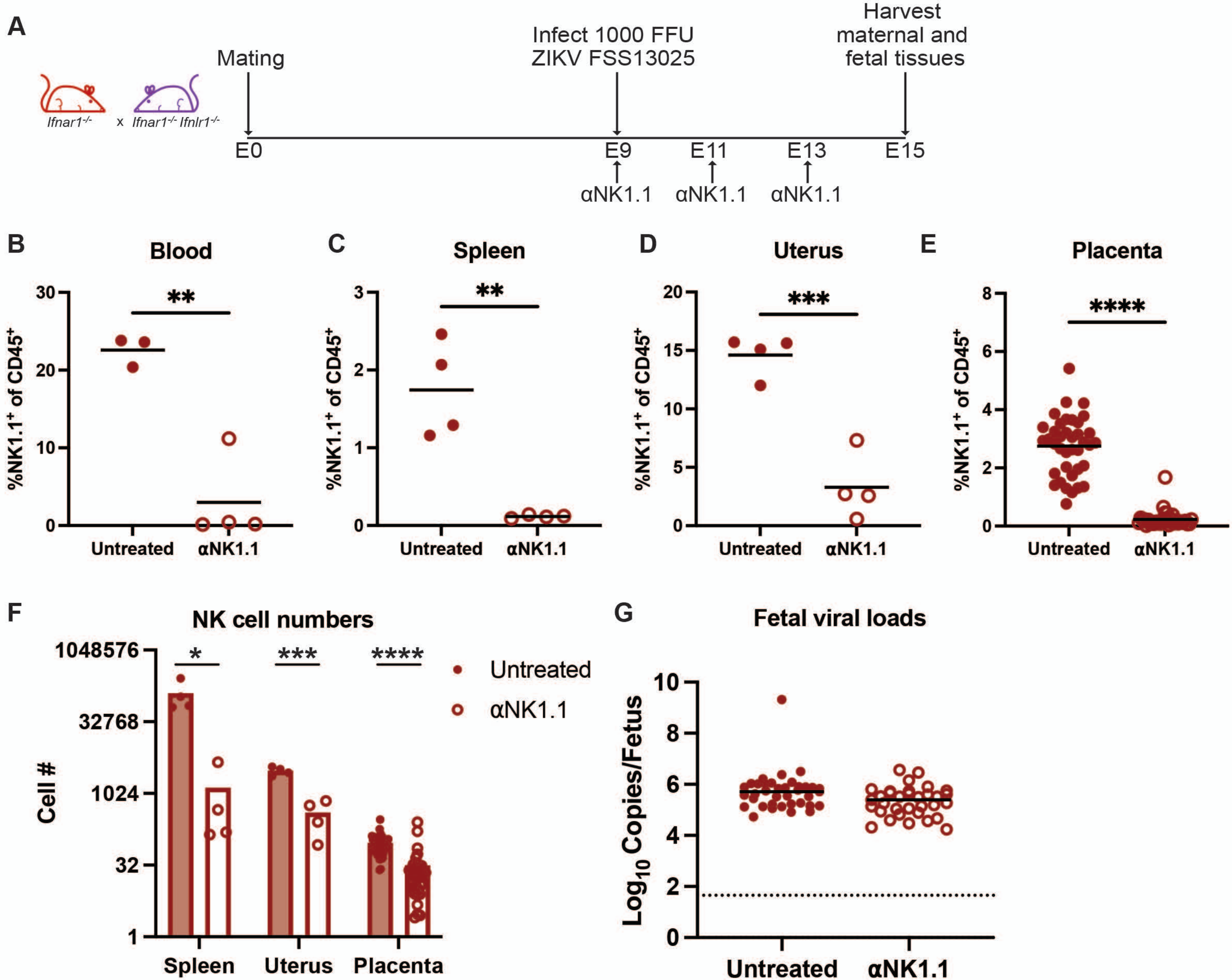
A
Mating
Infect 1000 FFU
ZIKV FSS13025
Harvest
maternal and
fetal tissues
Ifnar1-/-
x
Ifnar1-/- Ifnlr1-/-
E0
E9
E11
E13
E15
αNK1.1
αNK1.1
αNK1.1
B
Blood
C
Spleen
D
Uterus
E
Placenta
%NK1.1+ of CD45+
**
**
***
****
Untreated
αNK1.1
F
NK cell numbers
1048576
32768
1024
32
1
Cell #
*
***
****
Untreated
αNK1.1
Spleen
Uterus
Placenta
G
Fetal viral loads
Log10 Copies/Fetus
Untreated
αNK1.1

Figure S7
